# Genomic and Functional Characterization of Multidrug-Resistant *E. coli*: Insights into Resistome, Virulome, and Signaling Systems

**DOI:** 10.3390/antibiotics14070667

**Published:** 2025-06-30

**Authors:** Vijaya Bharathi Srinivasan, Naveenraj Rajasekar, Karthikeyan Krishnan, Mahesh Kumar, Chankit Giri, Balvinder Singh, Govindan Rajamohan

**Affiliations:** 1 CSIR-Institute of Microbial Technology, Sector 39A, Chandigarh 160036, India; 2 Biological Sciences Division, Academy of Scientific and Innovation Research (AcSIR), Ghaziabad 201002, India

**Keywords:** WHO critical threat, *E. coli*, antimicrobial resistance, One Health, CpxAR signaling, protein kinases

## Abstract

**Introduction:** Genetic plasticity and adaptive camouflage in critical pathogens have contributed to the global surge in multidrug-resistant (MDR) infections, posing a serious threat to public health and therapeutic efficacy. Antimicrobial resistance, now a leading cause of global mortality, demands urgent action through diagnostics, vaccines, and therapeutics. In India, the Indian Council of Medical Research’s surveillance network identifies *Escherichia coli* as a major cause of urinary tract infections, with increasing prevalence in human gut microbiomes, highlighting its significance across One Health domains. **Methods:** Whole-genome sequencing of *E. coli* strain ECG015, isolated from a human gut sample, was performed using the Illumina NextSeq platform. **Results:** Genomic analysis revealed multiple antibiotic resistance genes, virulence factors, and efflux pump components. Phylogenomic comparisons showed close relatedness to pathovars from both human and animal origins. Notably the genome encoded protein tyrosine kinases (Etk/Ptk and Wzc) and displayed variations in the envelope stress-responsive CpxAR two-component system. Promoter analysis identified putative CpxR-binding sites upstream of genes involved in resistance, efflux, protein kinases, and the MazEF toxin–antitoxin module, suggesting a potential regulatory role of CpxAR in stress response and persistence. **Conclusions:** This study presents a comprehensive genomic profile of *E*. *coli* ECG015, a gut-derived isolate exhibiting clinically significant resistance traits. For the first time, it implicates the CpxAR two-component system as a potential central regulator coordinating antimicrobial resistance, stress kinase signaling, and programmed cell death. These findings lay the groundwork for future functional studies aimed at targeting stress-response pathways as novel intervention strategies against antimicrobial resistance.

## 1. Introduction

Antibiotic resistance is a significant issue, wherein bacteria are capable of surviving even when exposed to the prescribed doses of antibiotics. This phenomenon highlights the diminishing efficacy of antibiotics in treating infections, posing a serious threat to public health worldwide [1]. The World Health Organization (WHO) has recently reported that antimicrobial resistance (AMR) is one of the leading causes of global mortality and economic loss [2,3]. AMR threatens the effectiveness of existing antibiotics, leading to prolonged illnesses, more deaths, and increased healthcare costs worldwide. Multidrug-resistant bacteria notably contribute to secondary infections, especially among vulnerable populations such as older adults, immunocompromised individuals, children, and pregnant women. These infections exacerbate existing health problems and further strain healthcare systems [4].

Misuse, over-the-counter sales without prescription, and the use of antibiotics as growth promoters in animal feed and bird supplements have facilitated their widespread entry into the food chain, contributing significantly to the spread of AMR [5]. The problem of AMR is no longer hidden from the public eye. Antibiotic awareness campaigns have brought the urgency of AMR to the forefront, mobilizing governments, healthcare providers, and the public toward immediate action [6].

The United Nations General Assembly High-Level Meeting on AMR, held on 26 September 2024, was a pre-planned, coordinated effort [7]. During this meeting, international stakeholders endorsed major revisions to the existing policy guidelines and introduced need-based criteria aimed at addressing this persistent and escalating issue. Another significant step in the fight against AMR was the release of an updated priority list by the WHO on 17 May 2024, followed by the Department of Biotechnology, India’s own update on 11 May 2021 [8,9,10]. This list categorizes pathogens based on their clinical significance, helping prioritize efforts in developing new treatments. Furthermore, the list clearly segregated clinically significant pathogens into categories of critical, high, and medium priority, providing a framework for researchers and funders to streamline their efforts and focus on the most urgent threats to public health [11].

According to the first quadripartite analytical report, in 2021, 118 out of 163 countries (72.3%) reported on the status of their AMR surveillance programs in humans. This shows growing global awareness and the importance of monitoring AMR trends across different regions [12]. Adoption of the WHO’s AWaRe antibiotic classification as primary treatment options by 59 out of 163 (36.2%) countries demonstrates a positive shift towards antibiotic stewardship [13]. This shift is a positive step in ensuring the responsible use of antibiotics to combat AMR [14].

In 2013, the Indian Council of Medical Research (ICMR) launched the Antimicrobial Resistance Surveillance and Research Network (AMRSN) program in India. This program annually publishes reports that track the changing patterns of antibiotic susceptibility and resistance across the country [15,16]. The tabulated data from the AMRSN highlights the evolving patterns of antibiotic susceptibility, as well as the levels of resistance observed in pathogens from the WHO priority list of superbugs. This information is crucial for understanding the shifting landscape of AMR [17,18,19]. In addition, ongoing monitoring of the Sustainable Development Goals (SDGs) adopted by various countries through their National Action Plans (NAPs) is crucial. Continuous, rigorous evaluation of implementation outcomes is crucial to identify gaps and guide improvements [20]. Data-driven insights are essential for defining standardized protocols to inform global policymaking. These insights can help inform best practices and shape the global response to AMR [21].

Additionally, significant efforts are being directed towards fostering industry–academic collaborations through the AMR seed grant scheme. This ensures robust participation from a diverse range of stakeholders, including researchers, clinicians, and industry leaders. While numerous efforts have been initiated and executed, the contributions of other critical pillars connected to the One Health approach, which exacerbate the AMR problem, must be explicitly integrated and addressed within multisectoral One Health strategies to comprehensively combat AMR [22].

Alarmingly, new strains of bacteria are emerging with high levels of both multidrug and cross-resistance. These strains present a significant challenge, as they are resistant to multiple classes of antibiotics, reducing the available treatment options. This situation is deeply concerning to clinicians, as there are no effective alternative treatments available for this widespread and growing crisis known as AMR [23]. The lack of viable therapeutic options is making it increasingly difficult to manage infections. The pipeline for new antibiotics is also alarmingly dry. Among the 97 antimicrobials currently in development, only 12 represent truly novel classes, with just four effective against WHO-designated critical priority pathogens [24].

This highlights the urgent need for innovation in antibiotic discovery. Therefore, identifying an ideal or conserved drug target that is effective across various species, serotypes, and pathogens is essential in the fight against AMR. Such a target could provide a unified strategy to tackle AMR on a global scale, preventing the further spread of resistant bacteria [25,26].

The development of a novel class of drug is critical, rather than merely identifying or designing modified or synthetic derivatives of existing anti-infective agents. This is necessary to ensure that we can address emerging resistant pathogens more effectively and reduce the likelihood of resistance development. New compounds must be resilient against resistance development, even at sub-inhibitory concentrations. They should not allow the development of resistance that could neutralize their mechanism of action. This strategy could potentially be the only feasible treatment option in the long term, as it would minimize the risk of resistance emergence [27,28].

The primary focus should be on fundamental investigative research into bacterial physiology, pathology, and persistence. This research will help us uncover the origins, roots, and progenitors of AMR, along with identifying the critical factors that drive the spread of resistance. Understanding these elements will provide insights into novel targets for drug discovery and development [29,30].

Surveillance studies spanning environmental and biological sources within the One Health framework will be invaluable in identifying the reservoirs and catalysts driving AMR transmission. These efforts will ultimately help eradicate the problem by targeting the root causes and preventing further spread [31]. Genome surveillance of atypical strains originating from plants, animals, and the human gut will serve a similar purpose. By sequencing these strains, we can uncover hidden, unique features of bacterial species, shedding light on their core virulome, resistome, and signaling systems, particularly stress protein kinases [32]. This will help us understand the historical evolution of these strains and identify any discrepancies or evolutionary changes in newly circulating variants or serotypes.

A key contributor to the growing AMR problem is the Gram-negative bacterium *Escherichia coli*, which is listed as a clinically significant pathogen on the WHO priority list [33]. This pathogen is frequently reported from clinical settings and various other sources, including in India, making it a critical target for AMR surveillance and intervention. Various strains of *E. coli*, such as enterotoxigenic (ETEC), enteropathogenic (EPEC), enteroinvasive (EIEC), enterohemorrhagic (EHEC), enteroaggregative (EAEC), and uropathogenic (UPEC), are capable of causing infections that range from mild to severe. These different pathotypes contribute to a wide range of clinical manifestations, further complicating the treatment landscape [34,35,36].

According to recent data from the ICMR-AMRSN, *E. coli* is identified as the predominant pathogen responsible for urinary tract infections (UTIs) [15,16]. This highlights its critical role in causing one of the most common bacterial infections worldwide. The pathogen’s resistance profile to carbapenems has alarmingly risen in recent years. This increase is concerning because carbapenems are often used as a last line of defense against multidrug-resistant infections [37,38].

The three key characteristics of *E. coli* that contribute to its classification as a nosocomial superbug are (i) its ability to persist on desiccated surfaces for extended periods, (ii) its tolerance to sterilization processes in clinical settings, and (iii) its remarkable capacity to acquire a wide array of resistance determinants and regulate their expression through sophisticated signal transduction mechanisms [39].

Importantly, *E. coli* is found ubiquitously across various environments, and the majority of these strains are involved in the spread and transmission of AMR in One Health settings. This is largely due to its robust genomic plasticity and adaptability, which allow it to thrive in diverse habitats and acquire resistance mechanisms. Furthermore, the multidrug-resistant to extensively drug-resistant nature of *E. coli* poses a significant challenge in One Health settings, where the available therapeutic options are limited. This restricts clinicians’ ability to effectively manage infections caused by resistant strains [40].

Interestingly, several studies have shown that commensal *E. coli* can act as a reservoir for AMR. These normally harmless strains of *E. coli* can harbor resistance genes and transfer them to pathogenic strains, exacerbating the spread of resistance. As a result, *E. coli* serves as an excellent model organism for studying the prevalence, diversity, mechanisms, and transmission of AMR. Its ability to exist as both a commensal and pathogenic organism makes it an ideal candidate for AMR research [41,42].

With rapid advancements in genome sequencing technologies and the increasing availability of comprehensive genomic datasets, detailed analysis of *E. coli* strains at the serotype and clonal levels has become indispensable. This high-resolution approach enables a more nuanced understanding of the ecological niches and clinical relevance of specific *E. coli* lineages, particularly in the context of AMR [43].

Notably, recurrent global reports of dominant *E. coli* sequence types (STs) such as ST131, ST10, and ST67—isolated from diverse sources including animals, birds, clinical specimens, and environmental reservoirs—underscore their persistent multidrug-resistant phenotypes. These predominant clones possess a remarkable capacity to acquire and disseminate a broad spectrum of antibiotic resistance genes, thereby posing significant challenges to AMR containment efforts [44]. Consequently, whole-genome sequencing (WGS) coupled with comprehensive genomic analyses of *E. coli* isolates from varied origins, including stool samples, is critical for precise tracking of resistance determinants and elucidating their role in the transmission dynamics of AMR [45].

Genome sequencing and detailed genotypic characterization are pivotal for enhancing surveillance frameworks and for gaining in-depth insights into the genetic diversity, evolutionary trajectories, and population structure of multidrug-resistant *E. coli* strains. Such information facilitates comparative analyses with other pathovars and serotypes, ultimately informing the design of targeted interventions and improved strategies for AMR management. In our comprehensive study of human microbiome isolates, we identified numerous *E. coli* strains corresponding to well-characterized serotypes. However, one gut-derived isolate was clonally distinct and exhibited multidrug resistance alongside phenotypic traits commonly associated with clinical strains. Due to these unique features, this isolate was prioritized for whole-genome sequencing (WGS) and in-depth characterization.

The *E. coli* isolate ECG015 from our laboratory collection was subjected to comparative analyses to elucidate its atypical characteristics relative to other *E. coli* strains derived from clinical and gut environments. This approach enabled us to uncover distinct attributes that may underpin its AMR and potential pathogenicity.

Among key regulators identified, the CpxAR two-component system (TCS) is well recognized as a key regulator of cell envelope stress responses [46,47]. Based on our in silico analyses, we hypothesize that CpxAR may exert broader regulatory control over additional cellular processes, including the expression of AMR genes such as those encoding resistance determinants and efflux pumps, as well as eukaryotic-like serine/threonine kinases, bacterial tyrosine kinases, and the MazEF toxin-antitoxin system (TA), which is implicated in programmed cell death (PCD) [48,49].

While experimental validation is required to substantiate these predicted regulatory roles, the identification of such a complex hierarchical regulatory network in *E. coli* is both novel and compelling. This report presents a comprehensive analysis undertaken to explore these insights and their potential implications.

## 2. Results

### 2.1. Genomic Characteristics of the Gut Isolate ECG015

To investigate the genomic features of the gut isolate *E. coli* ECG015, the strain was sequenced using the Illumina NextSeq 500 technology platform (San Diego, CA, USA). The draft genome sequence data is represented in 107 scaffolds, with a maximum contig length of 240,397 bp and a minimum of 56 bp, respectively. The GC content of the genome is 50.69%, and it encodes 4657 coding sequences, 73 tRNA genes, and seven rRNA genes.

The genome sequence was annotated using RAST, and analysis indicated 595 subsystems, which represent 63% coverage with a total of 2776 genes, of which 2622 are non-hypothetical and 154 are hypothetical genes. Further investigation showed the percentage of genes for different cellular metabolism subsystems, as depicted in Supplementary Figure S1A.

To investigate the functionality of the proteins, gene ontology was performed, and the analysis showed that 36.19% of genes are associated with molecular function, with the highest number of genes involved in DNA and ATP binding functions. Furthermore, the genes involved in various cellular components and biological processes account for 45.36% and 18.45%, respectively. In terms of cellular components, the highest number of genes are associated with the integral component of the membrane (20%), and in biological functions, 8.62% of genes are involved in DNA transcription, regulation, and repair processes. The genes associated with different functional components are shown in Supplementary Figure S1B.

**Figure 1:**
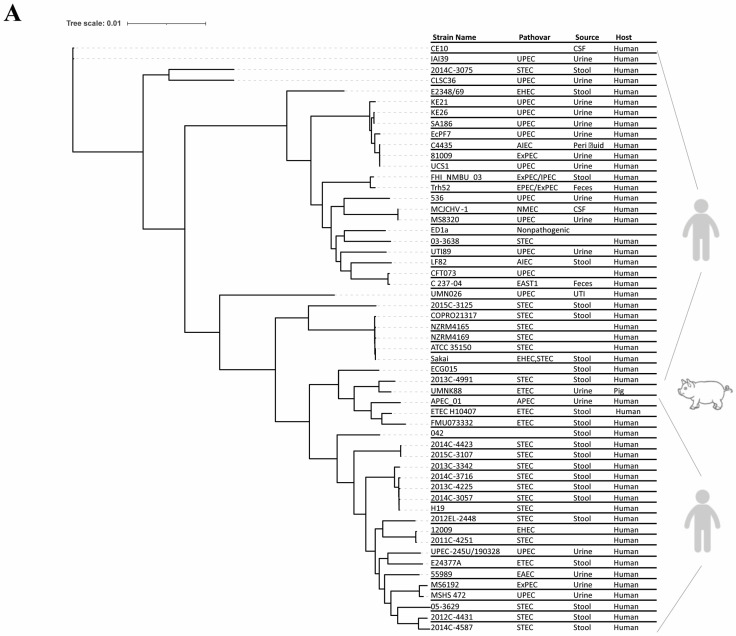

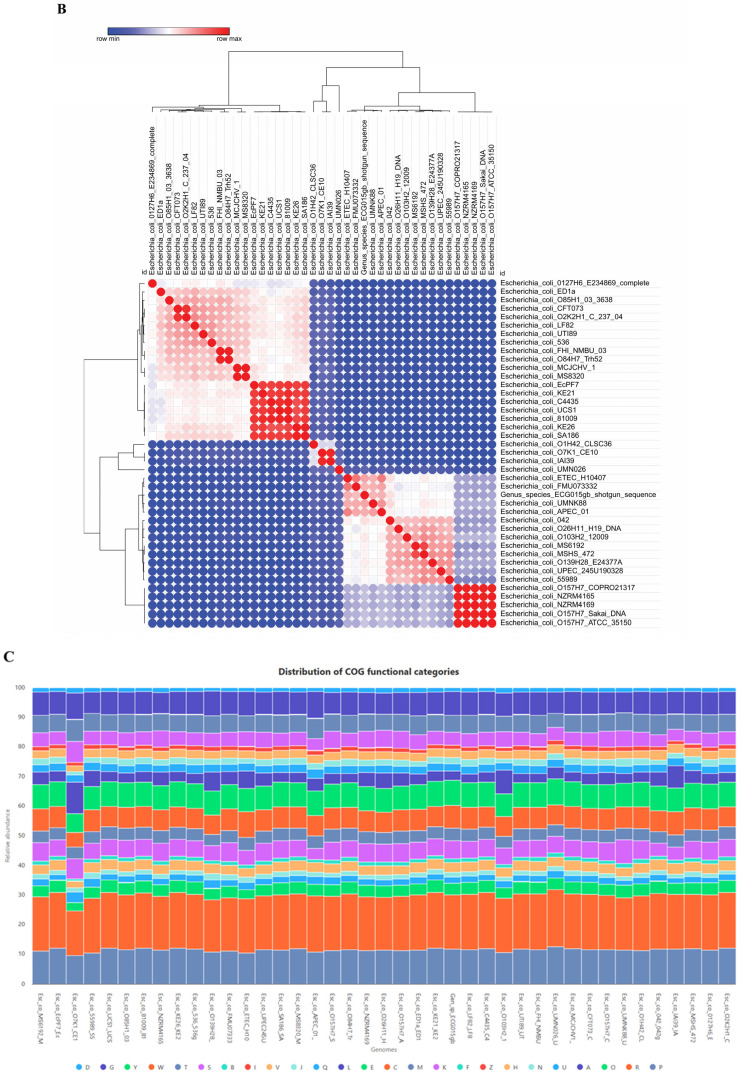

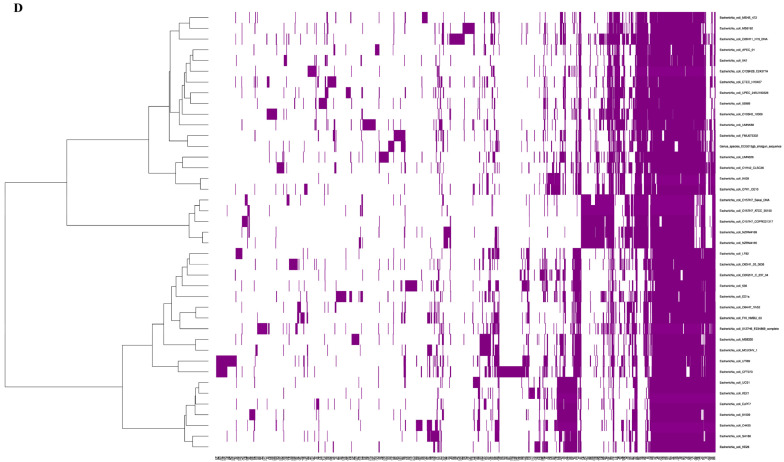
Genome analysis and phylogeny of *E. coli*: (**A**) whole-genome sequence-based phylogenetic trees of pathotype *E. coli* strains. The phylogenetic unrooted tree was constructed based on core genome single-nucleotide polymorphism (SNP)-based alignment using the maximum likelihood method. Various pathovars/pathotypes of *E. coli* strains were included, with *E. coli* Sakai serving as the reference genome in REALPHY (https://realphy.unibas.ch/realphy/) (accessed on 22 October 2024). The prominence of ECG015 in the tree was observed, and it clustered among human isolates in the selected set of *E. coli* pathovar strains. The whole-genome-based phylogenetic tree was constructed and plotted using iTOL. Details of the strains, genomes, and pathotypes are listed in Supplementary Table S1. (**B**) average nucleotide identity (ANI) score matrix for the *E. coli* strains. The ANI score heatmap represents the nucleotide similarity among the selected *E. coli* pathotype genomes. (**C**) Clusters of orthologous groups (COG) analysis of *E. coli* genomes. The distribution of the relative abundance of COGs in different *E. coli* pathotype genomes. The COG represents different colors (top to bottom) of functional annotation categories or terms. (**D**) Pan-genome analysis of the selected *E. coli* strains. The presence and absence of genes in the genomes of *E. coli* pathotype strains are represented in a clustered heatmap. Genes present in the genomes are shaded in color. The gene clusters and genomes were well-ordered with hierarchical clustering.

The phylogenomic analysis of selected *E. coli* pathovar genomes (*n* = 55) shows two major clusters, which interestingly diverge into further sub-clusters. The ECG015 strain clusters in a clade with closely related genomes, such as ETEC_H10407, 2013C-4991, FMU073332, UMNK88, and APEC_01, of both human and animal origin (Figure 1A). Likewise, the distribution of ANI scores among *E. coli* pathovars shows that the most similar genomes are grouped into different clusters (Figure 1B).

The functional classification of genes from different pathotypes of *E. coli* (*n* = 41) was analyzed using the COG database. The analysis showed the distribution of genes within COG categories for *E. coli* strains. The COG prediction for the *E. coli* ECG015 strain indicated a high number of genes involved in translation, ribosomal structure and biogenesis, amino acid transport, ion transport and metabolism, carbohydrate transport and metabolism, and energy production and conversion (Figure 1C). Overall, the ECG015 strain shows a relative abundance of genes involved in metabolism, cellular processing, and signaling.

Furthermore, the pan-genome analysis of *E. coli* (*n* = 41) was performed to examine the presence and absence of gene clusters representing core and dispensable genes (Figure 1D). To decipher the genes involved in various cellular metabolic pathways in ECG015, the Kyoto Encyclopedia of Genes and Genomes (KEGG) KAAS server was utilized for functional pathway annotation by BLAST +v2.16.0. This revealed the highest number of genes (313) involved in carbohydrate metabolism, followed by 142 for amino acid metabolism, 136 for metabolism of cofactors and vitamins, 107 for nucleotide metabolism, 103 for energy metabolism, 102 for glycan biosynthesis and metabolism, 56 for lipid metabolism, 43 for metabolism of other amino acids, and 16 for terpenoid and polyketide metabolism.

**Figure 2:**
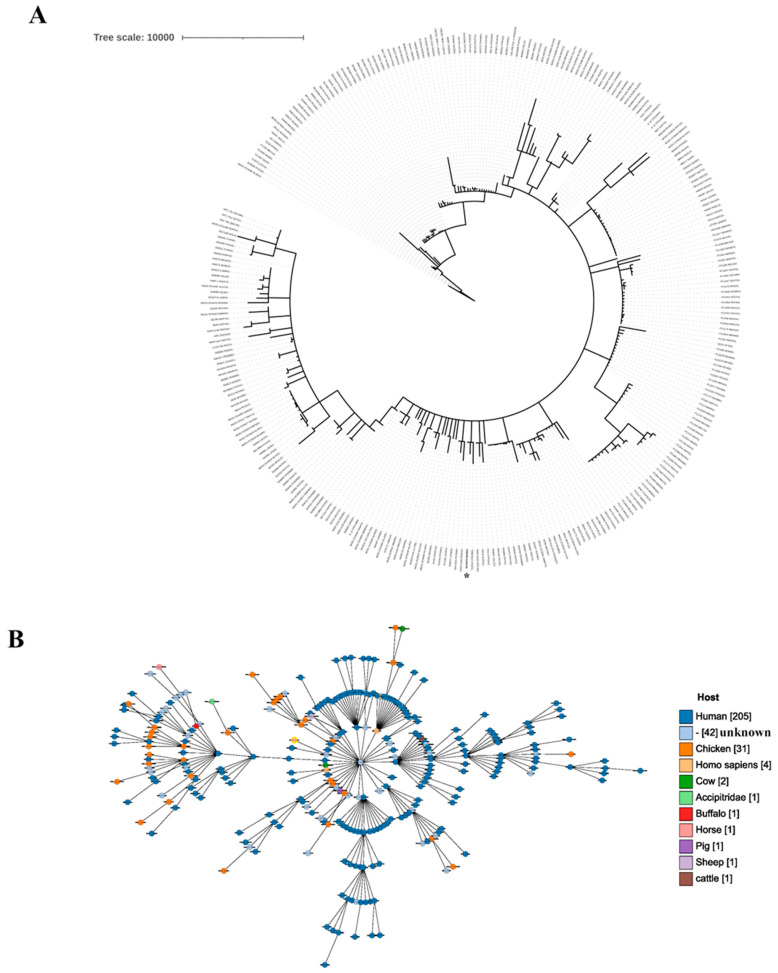
Phylogenetic representation of *E. coli* strains from India. (**A**) Phylogeny of whole-genome sequences of Indian *E. coli*. The circular phylogenetic tree based on core genome multilocus sequence typing (MLST) was constructed for ECG015 (*) along with 291 available Indian *E. coli* isolates from different hosts, using the BacWGSTdb server. The SNP-based phylogeny for Indian *E. coli* strains was generated with reference to the pandemic clone *E. coli* CD306 genome, which belongs to ST131 (CP013831). The strain and accession numbers are listed in the inner and outer rings of the tree. (**B**) Grapevine tree representation of ECG015 with Indian isolates from different hosts. The grape tree generated from whole-genome sequences illustrates the relationship between Indian *E. coli* isolates from various hosts. These isolates are clustered into distinct groups. The strain ECG015, highlighted with a yellow circle (*), is associated with human isolates and appears in the central cluster. The log-scale tree was generated, and the numbers in parentheses represent the number of Indian isolates from various hosts.

The SNP-based phylogenetic tree was constructed using available Indian *E. coli* genomes (*n* = 290) from various hosts (BacWGSdb, accessed in October 2024, May 2025). The tree was visualized using iTOL v7. Additionally, an MLST-based phylogenomic grape tree was constructed for the Indian *E. coli* strains, and the analysis revealed major clusters with closely related strains forming separate sub-clusters. The tree analysis showed the distribution of *E. coli* genomes from diverse hosts, with ECG015 clustering together with strains of human origin (Figure 2A,B).

### 2.2. Sequence Comparison of Etk and Wzc Tyrosine Kinases

Phosphorylation mechanisms in bacteria are primarily driven by two-component systems, which consist of histidine kinases and response regulators. Additionally, bacteria also harbor one-component signaling systems, which include serine/threonine, tyrosine, and arginine kinases. Genome analysis of the ECG015 strain revealed the presence of two types of bacterial tyrosine kinases, Etk and Wzc, alongside a serine/threonine kinase involved in phosphorylation signaling. We aim in future to further investigate these structurally and functionally distinct kinases due to their exclusive and non-redundant roles in regulating the capsule, biofilm formation, stress responses, virulence, and AMR. To this end, we generated bacterial tyrosine kinase (dispensable genes Etk and Wzc) and serine/threonine protein kinase (core) gene clusters for a variety of *E. coli* pathovar genomes. The comparative homologous gene clusters were visualized using Clinker to illustrate the core and dispensable kinase gene clusters among the selected *E. coli* genomes (Supplementary Figure S2A–D). To better understand the conserved and insertion regions within the tyrosine kinases Etk and Wzc, we performed a multiple-sequence alignment. The analysis revealed that the Tyr574, a key phosphorylation activation switch, along with its interacting residue Arg614, ATP-binding Walker motifs A, A’, B, and the C-terminal tyrosine (Y) cluster regions, are highly conserved across both Etk and Wzc kinases. Structural analysis further indicated that the folds of the Etk and Wzc kinase domains differ significantly from one another. The positively charged Arg-Lys (RK) cluster region, which is in close proximity to the C-terminal tyrosine (Y) clusters, plays a crucial role in the capsular polysaccharide export machinery, as previously described [50]. In addition, the signature sequence of the C-terminal Y cluster region was found to be evolutionarily conserved in both kinases (Supplementary Figure S3A). Multiple-sequence analyses of Etk and Wzc tyrosine kinases from selected Gram-negative bacteria exhibited 99% identity and 70% similarity to kinases from non-*E. coli* hosts.

Phylogenetic analysis of these homologous kinases resulted in the formation of two major clusters, segregating Etk and Wzc kinases distinctly (Supplementary Figure S3B). Similar findings were observed in the larger sequence analysis (Supplementary Figure S4A). Furthermore, homology modeling of Etk (blue) and Wzc (magenta), along with their structural superimposition, revealed key structural differences and similarities, shedding light on the intricacies of these kinases (Supplementary Figure S4B).

### 2.3. Prevalence of Virulence Genes in the Gut Isolate ECG015

The repertoire of virulence genes found in the isolate ECG015 was no different from those found in clinical strains, which is alarming. The virulence factors identified here include the following: arylsulfatase (A8A05_22485), major structural subunit of curlin (A8A05_08150), intimin-like adhesin FdeC (A8A05_13230), type-1 fimbrial protein subunit A (A8A05_07330), glutamate decarboxylase (A8A05_14800), hemolysin activation protein (A8A05_18660), increased serum survival lipoprotein (A8A05_20770), capsule biosynthesis protein (A8A05_04770), polysialic acid transporter (A8A05_00535), lipoprotein NlpI (A8A05_03580), shikimate transporter (A8A05_05945), tellurium ion resistance (*terC*) family proteins (A8A05_03205), and capsular polysaccharide transport protein YegH (A8A05_16450). Interestingly, we found proteinaceous adhesins in the genome of ECG015, known to play roles in biofilm formation, attachment to host cells for colonization, and bacterial pathogenesis.

The strain was screened against more than 500 adhesin genes reported for *E. coli* strains using the adhesiome R webserver. The analysis revealed the presence of 64 genes encoding adhesins, comparable to well-studied pathotypes of *E. coli*, which usually harbor 50–80 adhesion genes. These virulence factors are mostly involved in adherence, including *aatA*, AIDA-I type, Curli fibers (*csgABCDEFG*), *E. coli* common pilus (*ecp*) (c*pABCDER*), Hemorrhagic *E. coli* Pilus (*hcp*), Type 1 fimbriae, UpaG adhesion, and fimbriae-related genes such as *sfmA*, *D*, *F*, *H*, *yadL*, *M*, *N*, *V*, *ybgO*, *P*, *Q*, *F*, *ycbQ*, *ycbR*, *S*, *T*, *U*, *V*, *ydeQ*, *R*, *S*, *T*, *yeeJ*, *yehE*, *yfaL*, and *yraH*, *I*, *K* (Supplementary Figure S5, Supplementary Table S3).

Further analysis demonstrates that the strain belongs to adhesion cluster AA and fimbrial cluster F-C. Cluster AA is the largest and most variable cluster found in multiple pathotypes of *E. coli*, while fimbrial cluster FC represents STEC strains harboring *Stg* fimbriae. Overall, the analysis corroborates that since the strain possesses these virulence-associated genes, it may have pathogenic potential. Additionally, the pathogen finder analysis indicates that strain ECG015 is grouped among pathogenic *E. coli* genome clusters with mean score of 0.9711. The prediction range is 0 for non-pathogenic and 1.0 for pathogenic capacity, suggesting that gut *E. coli* strains from healthy individuals can harbor virulence and resistance determinants, positioning them as potential reservoirs for antimicrobial resistance and latent pathogenicity.

PHASTEST was used to predict the prophage sequences in the ECG015 genome. The results revealed the presence of four phage-associated gene regions (1 to 4) of which one is intact, two are incomplete, and one is questionable (Supplementary Figure S6). The intact prophage region is 26.6Kb long contains 38 total proteins (scaffold4|size224099) with GC% of 48.55% and located in the region from 35,146 to 62065. Of the two incomplete prophages, one is 20.2Kb long and contains 15 proteins (scaffold16|size98585) with GC% of 46.53% and the second one is 28Kb long with 26 proteins (scaffold1|size240509) and GC% is 48.52%. The questionable prophage is 25.9Kb in length and contains 28 proteins in scaffold14|size107478, with GC percentage of 45.86%.

### 2.4. Genes Associated with Antibiotic Resistance Found in the E. coli Isolate ECG015

The resistance mechanisms in bacteria are multifaceted and broadly classified into upregulated multidrug efflux pump extrusion, enzymatic alteration of antibiotics, drug-specific target gene mutations, and restricted outer membrane permeability. The dihydrofolate reductase gene *folA* (A8A05_05570) was found in scaffold 2xsize202585, and the cephalosporinase *ampC* β-lactamase gene (A8A05_01700), responsible for resistance to cefoxitin, cephalothin, and cefazolin, had mutations at eleven different positions (366/377—97.08%) relative to its homolog *E. coli* b4150.

The penicillin-insensitive transglycosylase and transpeptidase PBP-1C (A8A05_21815) had four missense mutations compared to its counterpart ECRM13514_3349. The D-alanine-D-alanine ligase gene *ddlB* (A8A05_05355), responsible for dipeptide formation required during cell wall biogenesis, had a missense mutation at the 194th position (G to D) relative to its homolog b0092. The polymyxin resistance protein PmrG (A8A05_09250) had three mutations: at position 15 (F to I), at position 136 (E to G), and at position 146 (R to S), along with mutations in the undecaprenyl-diphosphatase gene.

The *gyrA* subunit, involved in conferring quinolone resistance, had two mutations: at the 678th position (D to E) and the 741st position (D to E), while *gyrB* had no mutations. Similarly, the *parC* subunit had a mutation at the 470th position (Q to H), while *parE* had no mutations. The adenylate cyclase gene *cyaA* (A8A05_22460) had a mutation at the 142nd position (N to S) relative to b3806, suggesting a possible fosfomycin resistance behavior in this *E. coli* isolate.

In addition, the glycerol 3-phosphate, phosphate antiporter gene *glpT* (A8A05_09190), known to be responsible for elevating fosfomycin resistance, also had dual mutations at positions 409th (D to G) and 448th (E to K) residues relative to its sensitive homolog b2240. The hexose phosphate transport protein *uhpT* (A8A05_08665) had no mutations but was found intact in scaffold 6xsize153664.

### 2.5. Multidrug Efflux Pumps Identified in the Isolate ECG015

Predominant efflux pumps from the resistance-nodulation-division family were found enriched in this genome, namely *acrAB* (A8A05_12305, A8A05_12300), *acrEF* (A8A05_04075, A8A05_04080), and *mdtABC* (A8A05_16510, A8A05_16505, A8A05_16500).

The multidrug efflux pump membrane fusion lipoprotein AcrE has a single mutation at position 351 (G to A), while AcrF, the multidrug efflux pump RND permease (A8A05_04080), had a replacement at the 48th and 49th positions (GT to DI), along with mutations at positions 240 (F to L) and 770 (L to V).

The NorM homolog (A8A05_19945) from the MATE family, the drug antiporter EmrAB protein (A8A05_15920, A8A05_15925), and the broad-spectrum MdfA pump (A8A05_10980) from the MFS family were also identified.

The drug/metabolite transporter SugE (A8A05_01690) and EmrE (A8A05_02515) from the SMR family were also found intact in this gut isolate. The small multidrug resistance protein SugE had a mutation at position 105 (H to Q, A8A05_01690) compared to its homolog b4148. EmrE had mutations at three positions: at the 61st (A to G), 75th (S to L), and 100th (I to V) positions.

The ATP hydrolysis-utilizing membrane proteins known as ABC-type efflux pumps were also detected in our strain. The unconventional MacB multidrug efflux transporter, classified under the exporter class and a member of the Type VII superfamily in the ABC3 clade, accounted for the fourth type of tripartite efflux pump, *macAB* (A8A05_10625, A8A05_10620), and was found conserved in this gut *E. coli* ECG015 isolate. Unlike other homologs of MacB, bioinformatics analysis pinpointed the presence of an extended loop (skirting helical) in the cytoplasmic region and large periplasmic regions in MacB. These sequence variations indicate that MacB is idiosyncratic for this isolate and an ideal candidate for performing combined in silico structure–function experimental analysis.

The *E. coli* MacB protein is a non-canonical ATP-binding cassette (ABC) transporter that is not directly involved in substrate transport. Instead, it utilizes its cognate partner protein, MacA, to induce conformational changes in an outer membrane protein, thereby expelling antimicrobials from the cell. The conserved Walker A (P-loop) and Walker B (hydrophobic residues) motifs, located in the nucleotide-binding domain (NBD), are responsible for ATP binding and hydrolysis. ATP binding promotes dimer dissociation of MacB, generating mechanical energy that drives conformational changes in its transmembrane domain. These changes are transmitted to MacA and TolC to facilitate substrate export through the outer membrane. The transmembrane helices and periplasmic domain of MacB interact with the NBD, and this crosstalk is essential for signal relay to MacA and TolC, forming a functional tripartite efflux pump [51].

Sequence alignment of MacB from *E. coli* ECG015 and its homologs in selected Gram-negative bacteria and different *E. coli* strains is shown in Supplementary Figure S7(Ai,Aii), Supplementary Table S4. The analysis revealed the conservation of residues and a flexible disordered loop region residing on the cytoplasmic side. Additionally, the *E. coli* ECG015 MacB protein exhibited 53% identity and 99% similarity with *Acinetobacter baumannii*, 85% identity and 91% similarity with *Klebsiella pneumoniae*, and 48% identity and 69% similarity with *Aggregatibacter actinomycetemcomitans*.

A homology-based model of the ABC transporter MacB from *E. coli* ECG015 was created using the SWISS-MODEL server. The predicted model exhibited 99.69% sequence identity and coverage with the experimentally validated structure template 5nik.1.J, a macrolide export ATP-binding/permease protein MacB from *E. coli*. The MacB monomer of ECG015 was superimposed with MacB from *A. baumannii* and *A. actinomycetemcomitans*, as shown in Supplementary Figure S7(Bi–Biii). A flexible loop was found near the NBD region at the N-terminus of the protein; this region was not captured, possibly due to flexible conformations in the crystal structures of *E. coli*, *A. baumannii*, and *A. actinomycetemcomitans* (Protein Data Bank IDs: 5nik.1.J, 5GKO, and 5LJ7). In the absence of ATP substrate, the two NBD domains of the MacB dimer are physically separated, a state that is not favorable for ATP nucleotide binding or interaction [51].

The molecular drug-binding pocket analysis was performed using the predicted MacB model. The data indicated several potential binding pockets, with pocket P_0 displaying the highest drug score of 0.80 for druggability, and pocket P_6 exhibiting the lowest score of 0.55. The density map displayed the pockets (Supplementary Figure S7(Ci,Cii)), and the scores predicted for different binding pockets are shown in Table 1. Our ongoing studies on *macB* from *A. baumannii* point to a possible role for the flexible loop in mediating substrate specificity (manuscript in preparation).

Therefore, a combined bioinformatic and experimental approach will help identify the substrate-binding domains/residues and elucidate the role of the extended loop in the periplasmic and cytoplasmic regions of MacB. One such study is crucial in bacterial species, as the findings will help identify substrate-binding domains/residues and reveal the significance of the extended loop within the periplasmic and cytoplasmic regions of MacB. The findings will validate the importance of MacB as a drug target, which can be broadly exploited for novel drug discovery and immunization strategies, an integral focus of our ongoing research.

### 2.6. Signaling Systems Detected in the E. coli Strain ECG015

The PmrAB system that modulates antimicrobial peptide resistance with broad-spectrum specificity had sense mutations (P102L, E121K) along with the kinase PhoQ (A8A05_18830), which had mutations at positions 464 (E to D) and 482 (A to T), respectively, while its cognate regulator PhoP had alteration only at 44th (I to L) residue.

The two-component system PmrA and PmrB regulate lipopolysaccharide modifications with changes in lipid A.

The sensor kinase PmrB mutation P102L in the histidine kinase domain could stabilize a helical conformation, alter signal sensing, and promote constitutive phosphorylation of PmrA even without activating signals. The change from an acidic to a basic residue (E121K) in the kinase domain disrupts normal regulation, increases autokinase activity, and leads to the constant activation of PmrA. The mutations in PmrAB and PhoQP contributing to clinical resistance in bacterial pathogens are well documented [52,53,54,55,56,57,58,59]. Lin et al. demonstrated that the PmrB mutation P94L in clinical *E. coli* strains contributes to an increased colistin minimum inhibitory concentration (MIC) [60] and Alsahlani et al. showed that genetic alterations/mutations in PmrAB are critical for the development of colistin resistance [61]. Importantly, these mutations may play an important role in cellular survival adaptation and the evolution of resistance mechanisms by activating signaling proteins in a ligand/signal-independent manner.

PhoQP, a two-component system, is known to regulate multiple genes in response to environmental stimuli, such as low magnesium and antimicrobial peptides. PhoQ, a sensor histidine kinase, phosphorylates its cognate protein partner PhoP to regulate various genes under the PhoP regulon. Genetic mutations in PhoQP alter bacterial cellular metabolism, virulence, and AMR.

The mutation E464D in the catalytic domain and near the C-terminal region of PhoQ could modulate the autophosphorylation activity and lead to the constitutive activation of PhoP, making it less responsive to external signals like magnesium levels. Similarly, the mutation A482T will modulate ATP binding and promote increased kinase activity. Moreover, the PhoP mutation I44L in the receiver domain’s hydrophobic core, with the change of isoleucine to the slightly more flexible residue leucine, may contribute to stable and enhanced phosphorylation, leading to increased resistance. Significantly, these gain-of-function mutations might be involved in constitutive and enhanced PhoPQ signaling, which would increase bacterial cell resistance to antimicrobials [62,63,64].

CpxAR, a two-component system, responds to envelope stress and regulates multiple cellular functions, including cell membrane integrity and AMR. The CpxA sensor kinase protein at position 315 is mutated from S to G (A8A05_17055) compared to its homolog b3911. The S315 mutation, located in the kinase domain and responsible for ATP binding and autokinase activity, is likely to impair CpxA autophosphorylation and reduce CpxR activation. Previous studies have reported that kinase mutations reduce CpxR activation and lead to impaired envelope stress tolerance with increased beta-lactam susceptibility in *E. coli* [65,66,67].

Similarly, the repressor of the AcrAB-TolC efflux pump, AcrR, at position 28 is mutated from G to V (A8A05_12310) in the DNA-binding helix-turn-helix domain compared to its homolog b0464. The mutation reduces AcrR binding to the acrAB efflux pump, leading to the derepression of efflux pump expression. Studies have demonstrated that AcrR R45C in the HTH domain abolishes DNA binding and increases ciprofloxacin resistance. Additionally, a frameshift mutation in AcrR enhances tigecycline resistance in *Klebsiella pneumoniae* [68,69].

The gut isolate ECG015 also encodes the stress kinases, primarily the SrkA homolog (A8A05_17350) in its genome, along with its twin-threat tyrosine kinases, Wzc (A8A05_16435), and protein kinase, ETK or PTK protein tyrosine kinase (A8A05_15275).

### 2.7. Homology Modeling and Docking

The predominant signaling protein known to perform pleiotropic functions in bacteria is CpxR, for which the three-dimensional structure is not available. Therefore, a homology-based approach was employed in the SWISS-MODEL server to predict the structure of *E. coli* ECG015 CpxR, as shown in Supplementary Figure S8(Ai).

The structure exhibited 100% identity with the template P0AE88.1.A, a transcriptional regulatory protein CpxR, and the AlphaFold DB model of CpxR from *E. coli* K12. These results demonstrate the suitability of the modeled CpxR structure for molecular docking and analysis. The binding site prediction of CpxR revealed possible binding pockets, with pocket P_0 showing the maximum drug score of 0.8 for druggability, and pocket P_6 exhibiting the minimum score of 0.15. The density map depicts the pockets (Supplementary Figure S8(Aii)), and the scores predicted for different binding pockets are shown in Table 2.

The virtual screening of compounds against the CpxR protein, which is implicated in various cellular functions including AMR, identified more than 100 compounds with different binding abilities. The top-ranked six compounds were R428 (Bemcentinib, −9.7 kcal/mol), Proscillaridin (−8.4 kcal/mol), Ergotamine (−8.3 kcal/mol), Tozasertib (−8.2 kcal/mol), Etoposide-phosphate (−8.1 kcal/mol), and Etoposide (−8.1 kcal/mol), which displayed better binding potentials with binding energy values (kcal/mol).

Furthermore, the first two compounds were docked against CpxR using the fast DRH web server. The docking scores were −7.523 for R428 (Supplementary Figure S7(Bi)) and −5.255 for Proscillaridin (Supplementary Figure S8(Bii)). Additionally, the amino acids Leu38, Leu40, Leu41, Asp42, Ala65, Thr69, Gln68, Leu88, Leu92, Ala109, and Arg206 were identified as hotspot interface residues in the CpxR–R428 and CpxR–Proscillaridin complexes, respectively.

### 2.8. CpxR, the Master Regulator of AMR and Programmed Cell Death

Analysis of the promoter regions of identified antibiotic resistance genes in *E.coli* indicated the presence of probable CpxR binding sites [70], including AmpC β-lactamases (PMD86181, 46 bases from the start codon), penicillin-binding protein *pbpC* (PMD96415.1; A8A05_21815, 82 bases from the start of *yfhM*-*pbpC*), membrane fusion efflux protein *acrA* of the AcrAB complex (39 bases), efflux transporter *mdtJ* of the multidrug/spermidine efflux pump *mdtIJ* (46 bases), and the SMR efflux pump *sugE* (74 bases).

In addition to these resistance genes, the presence of putative CpxR binding boxes was observed in the promoter regions of signaling proteins, such as A8A05_16435 (*wzc*, PMD95089.1), A8A05_15275 (*etk*, PMD87305.1), and A8A05_17350 (*srkA*, PMD85406.1).

Molecular docking of DNA–protein complexes and their binding affinity was established with the identified CpxR-binding DNA sequences in the promoter regions of different genes (e.g., *wzc*, *srkA*, *acrA*, *mdtJ*, and *sugE*) and the homology-modeled CpxR protein in global and local docking programs, HDOCK and HADDOCK, respectively. The lowest docking score complex is considered the most reliable, and the parameters for each docked DNA–protein complex are shown in Table 3.

The residues in CpxR (ARG80, GLY81, SER82, GLU83, ARG86, VAL87, GLY173, VAL175, ARG178, GLU179, LEU190, THR191, PRO192, PHE193, ASP194, ARG195, ASP198, LYS219, LEU221, ARG222, GLY223, ARG224, LEU227) were selected as active residues for docking in the HADDOCK web server. The P*wzc*-CpxR interacting model, with the close proximity of residues (Gly81, Glu83, Pro192, Gly223, Arg222, Arg224) from the RING analysis, is depicted in Supplementary Figure S8(Ci–Ciii). The presence of CpxR binding sites on these promoters clearly suggests a potential regulatory influence and points to a concealed bacterial cascade that remains enigmatic in prokaryotes and requires experimental validation.

Salient in this network would be to understand under which physiological conditions these Ser/Thr/Tyr-specific protein kinases take over the functions of the cognate histidine kinase (CpxA) and use this central response regulator, CpxR, as its endogenous substrate. Undoubtedly, this is a vast area of research that demands significant R&D investment and systematic experimentation (knock out, complementation, gel shift, reporter assays, etc.), as findings from this would enable us to identify the ideal/conserved bacterial target that can be exploited for drug discovery and development.

Notably, the presence of CpxR binding boxes on promoter regions of proteins involved in controlling bacterial programmed cell death (PCD) is particularly concerning. First, its presence on the MazF toxin is known to have a role in triggering PCD in bacteria; second, its presence on the metabolic enzyme glucose-6-phosphate dehydrogenase (Zwf) is known to provide the extracellular death factor required to trigger PCD via the *mazEF* toxin-antitoxin system; third, its presence on the PCD antagonist kinase SrkA gene, and lastly on the PCD-triggering kinase Wzc, is alarming.

The presence of CpxR binding sites on promoters of stress kinases SrkA and Wzc, along with the genes involved in AMR and programmed cell death, clearly highlights the central role played by CpxAR as the global switch in bacteria. This system not only controls pathology, physiology, persistence, metabolism, and antibiotic resistance, but is possibly also the “Bacterial Cell Fate Regulator”. Overall, the predicted CpxR binding sites in the promoter regions of various genes suggest possible regulation by CpxAR signaling proteins. However, further experimental binding and interaction studies and validation remain to be evaluated.

Thus, the cell envelope system, CpxAR, has the mechanism to sense and adapt to changes in the environment to which the pathogen is exposed, possibly deciding the fate of the bacteria depending on cell density and host physiological stress conditions. What is disturbing is the presence of the same set of virulence and signaling proteins in a human gut isolate, similar to that of clinical strains. The analysis presented here undoubtedly converges onto the central dogma that “Human Gut Microbiota is the Reservoir of Antibiotic Resistance Genes”. Thus, periodic cohort-specific surveillance One Health approach based, correlated with treatment protocols, is essential.

## 3. Discussion

The rise of MDR infections represents a critical global health challenge, with Gram-negative pathogens such as *E. coli* at the forefront. In 2019, AMR-associated deaths were estimated at 1.27 million directly due to resistant infections and 4.95 million overall; *E. coli* alone was responsible for the highest number of deaths among bacterial pathogens [71,72]. *E. coli* bloodstream infections resistant to third-generation cephalosporins increased from approximately 75.1% in 2017 to 86.8% in 2020 in India [73,74].

These organisms increasingly evade treatment as traditional antibiotics lose efficacy, placing immense pressure on public health systems. Addressing this threat requires the development of innovative anti-infective strategies that go beyond conventional antibiotic approaches. In particular, studying emerging strains from human microbiome, animal, and environmental reservoirs can reveal the complex regulatory pathways that underlie resistance, virulence, and persistence [75].

Understanding these molecular mechanisms is crucial for identifying novel therapeutic targets and gaining insights into how *E. coli* adapts to antibiotic pressure. Such knowledge may ultimately help circumvent current treatment limitations and lead to more effective infection control strategies.

The increasing ineffectiveness of existing antibiotics underscores the need for precision-targeted strategies. By dissecting the intricate signaling pathways involved in *E. coli*’s survival, virulence, and resistance, we can identify new molecular targets for intervention. These insights offer the potential to develop pathway-specific therapeutics that block critical bacterial processes without promoting resistance.

*E. coli* remains a major cause of hospital-acquired infections, particularly in UTIs and bloodstream infections [76]. Developing drugs that interfere with key adaptive mechanisms in this pathogen could significantly improve patient outcomes and help reduce the global burden of AMR.

This study presents an in-depth genomic analysis of *E. coli* strain ECG015, a human gut isolate. Using advanced sequencing technologies and bioinformatics pipelines, we identified a broad repertoire of genes constituting the strain’s resistome (antibiotic resistance genes), effluxome (efflux pump-associated genes), and virulome (virulence determinants). Additionally, mutations potentially enhancing virulence and immune evasion were found, shedding light on the pathogen’s adaptability and persistence in host environments.

These genomic features underscore the strain’s capacity to thrive in diverse settings, from commensal gut environments to sites of active infection. The presence of genes associated with metabolic flexibility, immune modulation, and stress tolerance highlights the multifaceted strategies *E. coli* employs to colonize hosts and evade therapeutic interventions.

Though two-component systems are numerous compared to one-component systems in bacterial genomes [77], these two components likely have specialized functions Through detailed promoter analyses, we identified multiple predicted binding sites for the two-component regulatory system CpxAR within upstream regions of key resistance, metabolic, and TA system genes. These findings suggest that CpxAR may function as a global regulator, integrating diverse environmental signals to modulate gene expression during stress.

Mutations in the CpxAR such as activating point mutations in *cpxA*, gene deletions, or constitutive CpxR activation have been shown to enhance antimicrobial resistance in *E. coli* and *Salmonella enterica* by upregulating efflux pumps (AcrD), downregulating porins (OmpF), and increasing resistance to β-lactams, aminoglycosides, and fosfomycin [47,66,78]. The mutations identified in this study and their influence on the overall functional regulatory pattern of CpxR signaling require systematic investigation. Our findings strongly suggest that the CpxAR system possibly functions as a regulator of efflux systems in *Escherichia coli*, modulating not only proton motive force-dependent efflux genes (such as those of the resistance-nodulation-division family), but possibly ABC-type (ATP-binding cassette) transporters as well.

Of particular note is the potential role of CpxAR in regulating stress kinase-transforming proteins, which facilitate bacterial adaptation to hostile conditions, including antibiotic exposure and host immune attack. This positions CpxAR as a key coordinator of survival and persistence pathways in *E. coli*.

Our findings support the hypothesis that CpxAR serves as a molecular switch, controlling adaptive responses in *E. coli* under various external stressors, ranging from antibiotic exposure to immune pressure and environmental fluctuations. The broad distribution of predicted CpxAR-binding motifs upstream of stress-associated genes reinforces its potential as a master regulator.

CpxAR may also influence the expression of TA modules, which mediate stress tolerance, dormancy, and PCD. These TA systems may function as intracellular “decision-making circuits,” enabling *E. coli* to toggle between persistence, adaptation, or cell death depending on environmental conditions.

We propose that CpxAR balances survival and self-destruction in response to the severity and duration of stress, particularly under varying antibiotic concentrations. Under high antibiotic pressure, CpxAR may enhance resistance and persistence pathways. Conversely, under sub-lethal or prolonged low-level stress, it may activate death pathways to eliminate compromised cells, thereby preserving population fitness.

Our data indicate the presence of a conserved signaling network in *E. coli* that converges on CpxAR as a central regulatory hub. This system integrates stress response, metabolism, and virulence, key facets of bacterial fitness, suggesting a broader evolutionary role in prokaryotic survival and pathogenesis.

By coordinating these essential functions, CpxAR likely enhances *E. coli*’s ability to survive fluctuating conditions within hosts and external environments, including during infection and antibiotic treatment. This reinforces its relevance as a potential master regulator in bacterial adaptation and disease progression.

Given its central role in regulating bacterial stress responses and survival pathways, CpxAR emerges as a promising therapeutic target. Disrupting its function could dismantle core adaptive mechanisms, rendering *E. coli* more susceptible to host defenses and antibiotic treatment.

To fully substantiate this hypothesis, further experimental studies are needed to characterize CpxAR’s regulatory roles under different stress conditions. Validating its function as a stress-sensing decision-maker would establish CpxAR as a high-value target for antimicrobial drug development, our ongoing research mandate.

This study elucidates a potentially conserved signaling architecture in *E. coli*, centered on the CpxAR two-component system, that governs resistance, virulence, and survival. Our integrative genomic and promoter analyses reveal a new dimension of stress adaptation in bacterial pathogens and provide a compelling rationale for targeting regulatory networks, rather than individual resistance genes, as a next-generation antimicrobial strategy.

## 4. Materials and Methods

### 4.1. Genome Draft Sequence, Annotation, and Analysis

The *E. coli* strain ECG015 used in this study for genome sequencing and comparative analysis was obtained from the lab collection as part of an institutional network study. Chromosomal DNA was isolated using the ZR Bacterial/Fungal Miniprep DNA Kit (Zymo Research Corp., Irvine, CA, USA) and quantified using a NanoDrop spectrophotometer (Thermo Scientific, Waltham, MA, USA). Draft genome sequencing was performed using paired-end technology on the Illumina NextSeq 500 platform, as previously described [79]. Briefly, de novo assembly of adapter-free, error-corrected data was performed using the SPAdes v3.1 assembler (St. Petersburg genome assembler) Scaffolding of pre-assembled contigs using NGS paired-read data and gap closure was carried out with SSPACE v2.0. Sequence annotation was performed using the NCBI Prokaryotic Genome Annotation Pipeline, and novel open reading frames were identified using NCBI BLASTp v2.14.1. The draft genome was compared with reference genomes using CONTIGuator 2.7.5. Functional annotation was further supported by the RAST server, pathway analysis by KASS (KEGG Automatic Annotation Server), and identification of unique proteins using PATRIC server (https://www.bv-brc.org (accessed on 10 March 2024)).

The genome sequence of *E. coli* strain ECG015 was compared and its phylogenetic distribution analyzed against various *E. coli* pathotype genomes (Supplementary Table S1) using REALPHY (https://realphy.unibas.ch/realphy/ (accessed on 22 October 2024)). Additionally, strain ECG015 was compared with 291 *E. coli* isolates reported from India, and a core genome multilocus sequence typing (cgMLST) tree was constructed, rooted in the reference strain *E. coli* CD306 (GenBank: CP013831, ST131), using BacWGSTdb (http://bacdb.cn/BacWGSTdb (accessed on 20 October 2024)) (Supplementary Table S2) [80]. Based on cgMLST profiles of Indian *E. coli* isolates, a minimum spanning tree and graph tree were generated and analyzed using BacWGSTdb 2.0 and iTOL v7 [80,81].

Pan-genome analysis for core and dispensable genes, ANI-based genome clustering, COG distribution, and genome cluster alignment with Clinker was performed using Pan-Genome Explorer [82]. Antimicrobial resistance (AMR) determinants and phage elements were identified using the Comprehensive Antibiotic Resistance Database (CARD, https://card.mcmaster.ca/analyze/rgi (accessed on 2 February 2024)) and PHASTEST (https://phastest.ca/ (accessed on 22 January 2025)), respectively [83,84,85]. ResFinder was used to predict AMR genes, VR2 profiles were used to identify the abundance of virulence genes, and Mobile Finder was used to detect associated mobile genetic elements [83,84].

Adhesion genes were identified using AdhesiomeR (https://adhesiomer.quadram.ac.uk/ (accessed on 13 May 2025) or https://biogenies.info/adhesiomeR (accessed on 13 May 2025) or https://github.com/ksidorczuk/adhesiomeR (accessed on 13 May 2025)), and gene presence/absence heatmaps were generated using web based tool Morpheus (https://software.broadinstitute.org/morpheus (accessed on 13 May 2025)) [86].

Pathogen finder (https://genepi.dk/pathogenfinder (accessed on 15 May 2025)) was used to predict the pathogenic capacity of the ECG015 strain [59]. Comparison of virulence genes, mobile transposons, and chromosomally encoded resistance genes was conducted using the PATRIC server (https://www.bv-brc.org (accessed on 14 May 2025)). Promoter sequence analysis was performed using the Softberry tool [87,88].

The draft genome sequence of *E. coli* ECG015 has been deposited in NCBI GenBank under the accession number MADI00000000.

### 4.2. Multiple-Sequence Alignment and Phylogenetic Tree

Protein tyrosine kinase sequences of Etk and Wzc, along with their homologues, were retrieved from the PATRIC and UniProt databases from various *E. coli* pathotypes. A total of 19 Wzc and 10 Etk protein sequences were used for multiple-sequence alignment, including the Etk and Wzc sequences from the *E. coli* ECG015 strain. The alignment was performed using Clustal Omega v 1.2.4 [89]. The resulting alignment file was used in ESPript 3 to generate a multiple-sequence alignment annotated with secondary structure information, using the corresponding protein model protein data bank (PDB) file. A phylogenetic tree was subsequently constructed and visualized using iTOL v7 [90]. To further investigate the phylogenetic distribution of these protein tyrosine kinases, over 2000 Etk and Wzc homologous sequences from *E. coli* genomes were retrieved from the PATRIC database. These sequences were aligned using Clustal Omega, and the resulting phylogenetic tree was visualized in iTOL.

### 4.3. Homology Modelling, Molecular Docking, and DNA–Protein Complex

The protein sequences of tyrosine kinases Etk, Wzc, and the response regulator CpxR from the *E. coli* ECG015 genome were retrieved from NCBI (Etk: WP_102203664.1/A8A05_RS19340; Wzc: WP_102203533.1/A8A05_RS02845; CpxR: WP_001033722.1/A8A05_RS23535) for homology-based structural modeling using the SWISS-MODEL web portal [91].

Homology models were generated using SWISS-MODEL, which showed 99.86% sequence identity with the template Q8XC28.1, a tyrosine-protein kinase Etk model from AlphaFold DB (ETK_ECO57, *E. coli* O157:H7), and 99.44% identity with the template P76387.1, a tyrosine-protein kinase Wzc model from AlphaFold DB (WZC_ECOLI, *E. coli* K12).

A docking-based virtual screening approach was employed to identify potential binding compounds targeting CpxR using the MTiOpenScreen platform [92].

Compounds with high-confidence scores from the initial screen were selected for molecular docking using AutoDock Vina through the FastDRH web server (http://cadd.zju.edu.cn/fastdrh/overview (accessed on 27 October 2024)) [93]. Docking scores, binding poses, and hotspot residues were analyzed for the top-ranked ligand–CpxR complexes. Binding pocket prediction for CpxR was performed using the Proteins Plus server (http://proteinsplus.zbh.uni-hamburg.de (accessed on 3 November 2024)) [94].

To explore the CpxR–DNA interaction, consensus CpxR DNA-binding sequences were selected from the promoter regions of various genes in the ECG015 genome. These DNA fragments, along with the modeled CpxR protein, were used to generate protein–DNA complex models via HDOCK and HADDOCK servers [95,96]. Residues involved in protein–DNA interactions were analyzed using the Residue Interaction Network Generator (RING) [97]), and structural visualizations were created using PyMOL v2.5.5.

## 5. Conclusions

This study highlights the presence of resistance determinants, virulence genes, and protein kinases in the gut isolate. In addition, in silico analysis confirms the presence of CpxR binding sites on crucial promoter regions, emphasizing the existence of a hidden hierarchical network. Cell envelope proteins, such as CpxAR, maintain membrane integrity by sensing periplasmic disturbances and enabling necessary alterations for bacterial survival and colonization. CpxAR remains the principal signaling system that initiates and regulates the protein stress kinase apparatus, which is directly involved in the emergence of AMR, cellular adaptation under selective pressure, and persistence on abiotic surfaces [98].

Investigating the multifaceted and dynamic functions of the CpxAR system could prove crucial, as this signaling pathway is likely a central regulator of bacterial physiology. It is hypothesized that CpxAR’s role extends far beyond merely responding to stress; it may integrate a wide range of cellular activities, enabling bacteria to adapt to varying environmental conditions. This system might regulate gene expression, modulate cellular metabolism, control protein folding, and manage responses to external stresses, each of which contributes to bacterial survival and pathogenicity. Furthermore, it is plausible that CpxAR governs cell fate decisions, potentially determining whether a bacterium enters dormancy, persists, or undergoes PCD in response to environmental cues.

Given its involvement in these fundamental processes, CpxAR is anticipated to be a highly conserved and promising drug target. Our ongoing research hypothesizes that gaining a more comprehensive understanding of CpxAR’s pleiotropic functions could uncover novel insights, leading to the development of targeted therapies aimed at inhibiting its activity. Such approaches may prove effective in combating AMR and improving clinical outcomes in bacterial infections.

## Figures and Tables

**Table 1:** The prediction of binding sites in *E. coli* ECG015 MacB using protein plus server. The top six DoGSite Scorer predicted binding pockets (P_0 to P_6) for MacB are shown below with degree of score; the higher the score is estimated to be, the more druggable the binding pocket.

Name	Volume (Å)	Surface (Å)	Drug Score	Simple Score
P_0	1970.39	2486.25	0.809533	0.61
P_1	898.95	1030.4	0.830871	0.58
P_2	519.03	784.57	0.815272	0.32
P_3	472.18	689.34	0.76657	0.24
P_4	396.54	782.71	0.745362	0.33
P_5	374.19	711.25	0.619852	0.21
P_6	363.16	596.45	0.556378	0.17

**Table 2:** The prediction of binding sites in CpxR from ECG015 strain using protein plus server. The top six DoGSite Scorer predicted binding pockets (P_0 to P_6) for CpxR are shown below with degree of scores; the higher the score is estimated to be, the more druggable the binding pocket.

Name	Volume (Å)	Surface (Å)	Drug Score	Simple Score
P_0	1117.44	1388.63	0.8	0.6
P_1	209.28	287.32	0.45	0.0
P_2	191.36	488.81	0.46	0.15
P_3	185.15	253.78	0.27	0.0
P_4	138.24	265.49	0.35	0.05
P_5	133.95	314.54	0.23	0.0
P_6	121.41	199.14	0.15	0.0

**Table 3:** The top ranked DNA–protein interaction model based on HDOCK analysis and docking score.

Name	Docking Score	Confidence Score	Ligand Rmsd (A)	LG Score	MaxSub
*acrA1*-CpxR	−215.11	0.7862	34.10	6.100	0.516
*acrA2*-CpxR	−222.27	0.8093	25.12	6.100	0.516
*AcrD*-CpxR	−209.86	0.7680	65.49	6.100	0.516
*SrkA*-CpxR	−208.93	0.7647	67.59	6.100	0.516
*Wzc*-CpxR	−219.20	0.7996	34.56	6.100	0.516
*sugE*-CpxR	−221.04	0.8055	39.87	6.100	0.516
*mdtJ1*-CpxR	−212.14	0.7761	46.48	6.100	0.516
*mdtJ2*-CpxR	−221.18	0.8059	350.99	6.100	0.516

## Data Availability

All data included in this study. The draft genome of *E. coli* ECG015 was submitted in GenBank NCBI with the accession number MADI00000000.

## Supplementary Materials

The following supporting information can be downloaded at https://www.mdpi.com/article/10.3390/antibiotics14070667/s1, Table S1: The details of strain, genome and pathotype of *E. coli* isolates. Table S2: Details of Indian *E. coli* strains. Table S3: Predicted virulence factors in E.coli genome ECG015. Table S4: The details of organisms and protein locus numbers. Figure S1: A. Distribution of subsystems in *E. coli* ECG015 genome. The RAST server was used to predict the subsystems and associated genes in different subsystem categories in ECG015 genome. B. Gene ontology annotation of *E. coli* ECG015 genome. The functional annotations of predicted genes in ECG015 genome were achieved through gene ontology (GO) annotations. The predicted genes were represented in three GO categories, molecular function, cellular component, and biological process. Each category comprises multiple genes encodes for different cellular functions. Figure S2: Comparative gene cluster alignment and visualization in *E. coli* genomes. Comparison of gene cluster alignments of different *E. coli* genomes along with *E. coli* ECG015 (indicated with black closed circle). Pan-genome explorer (https://panexplorer.southgreen.fr (accessed on 3 November 2024)) based clinker visualization exhibit comparison of gene clusters of *Etk* (CLUSTER0379 tyrosine protein kinase)—SF2A, *Wzc* (CLUSTER6477 tyrosine protein kinase)—SF2B, *srkA* (CLUSTER2224 stress response kinase SrkA)—SF2C and *prkA* (CLUSTER9004 PrkA family serine protein kinase)—SF2D along with its flanking genes in ECG015 and other *E. coli* pathotype genomes. The genome accession numbers are mentioned in Supplementary Table S1. Figure S3: Multiple-sequence alignment and phylogeny of *E. coli* protein tyrosine kinases Etk and Wzc. A. Multiple-sequence alignment of protein tyrosine kinases from selected *E. coli* pathovars. The homologs of *E. coli* Etk and Wzc from various *E. coli* pathovars, *Shigella dysenteriae*, *Klebsiella pneumoniae*, and *Acinetobacter baumannii* were selected for multiple-sequence alignment using CLUSTAL Omega and formatted using the ESPript server. The *E. coli* Wzc homologs used for alignment include the following strains: ECG015 (A8A05_16435); FMU073332-ETEC (BLJ80_15220); LF82-AIEC (K8B90_14415); FHI_NMBU_03-ExPEC/IPEC (BXO92_00085); E2348/69-EHEC (E2348C_2202); Sakai-EHEC,STEC (BAB36288.2); C237-04-EAST1 (N7L43_09425); MCJCHV-1-NMEC (DPQ29_09290); MS6192-ExPEC (MS6192_02285); 2013C-4225-STEC (C6997_RS23570); 2015C-3107-STEC (C6N21_RS05990); 05-3629-STEC (C6P66_RS20030); 81009-ExPEC (DNNLJILF_02338); KE26-UPEC (O2W55_14155); SA186-UPEC (CHQ92_03580); UCS1-UPEC (LDM94_11940); CLSC36-UPEC (FK541_10155); UTI89-UPEC (MKD43_11185); *Klebsiella pneumoniae* (SSG20669.1); and *Acinetobacter baumannii* (SST05051.1). The *E. coli* Etk homologs used in this study are ECG015 (A8A05_15275); FMU073332-ETEC (WP_000208639.1); FHI_NMBU_03-ExPEC/IPEC (WP_104934141.1); E2348/69-EHEC (WP_000208660.1); Sakai-EHEC,STEC (NP_309164.1); MS6192-ExPEC (WP_000208650.1); 2013C-4225-STEC (WP_000208650.1); 2015C-3107-STEC (WP_000208650.1); 05-3629-STEC (WP_000208650.1); *Shigella dysenteriae* (EFP9419961.1); and *Klebsiella pneumoniae* (CDK77046.1). The accession numbers for each strain are provided in the parentheses. The *E. coli* pathotypes are indicated before each strain name, and the accession numbers are provided in brackets. In the alignment, the highly similar regions are highlighted with a colored background, conserved residues across groups are boxed, and the consensus sequences are displayed below the alignment. B. Phylogenetic relationship of *E. coli* protein tyrosine kinases. A phylogenetic tree was generated using iTOL for the different Etk and Wzc proteins, which were represented in the multiple-sequence alignment. The Etk and Wzc proteins are segregated into two prominent clusters. Figure S4: Phylogenomic analysis of *E. coli* tyrosine kinases. A. Phylogenetic distribution of Etk and Wzc from *E. coli* strains. The Etk and Wzc protein tyrosine kinases from over 1000 different *E. coli* strains were selected for this study from the Bacterial and Viral Bioinformatics Resource Center (PATRIC) *E. coli* genome datasets. The sequences were aligned using CLUSTAL Omega, and rectangular phylogenetic tree was constructed in iTOL. The Etk and Wzc protein tyrosine kinases were found to be distantly placed and clustered distinctly in the phylogenetic tree. B. Homology models of *E. coli* tyrosine kinase proteins Etk and Wzc. Protein homology models for Etk and Wzc were generated using the respective protein sequences from the *E. coli* ECG015 genome on the SWISS-MODEL server (https://swissmodel.expasy.org (accessed on 26 October 2024)). The generated structural models for Etk and Wzc exhibited 99.86% sequence identity with template Q8XC28.1, a tyrosine-protein kinase Etk AlphaFold DB model (ETK_ECO57, *Escherichia coli* O157:H7), and 99.44% identity with template P76387.1, a tyrosine-protein kinase Wzc AlphaFold DB model (WZC_ECOLI, *Escherichia coli* K12), respectively. The modelled proteins, Etk (in blue) and Wzc (in magenta), along with their superimposition, were visualized using PyMOL. Figure S5: Distribution of adhesins in *E. coli* strains. Heatmap showing the distribution of adhesin genes in ECG015 strain and other *E. coli* pathotypes. The *E. coli* genomes were BLAST searched against reported adhesins and fimbriae systems in adhesiomeR (https://adhesiomer.quadram.ac.uk/app/adhesiomeR (accessed on 13 May 2025)). A heatmap of hierarchically clustered genes with a dendrogram was generated from adhesiomeR data for *E. coli* genomes in Morpheus (https://software.broadinstitute.org/morpheus (accessed on 13 May 2025)). Blue and red color in the heatmap indicates absence and presence of genes. Figure S6: Prediction of prophage sequences in *E. coli* ECG015 strain. The ECG015 genome was analyzed with PHASTEST for the presence of prophage sequence; analysis resulted in one intact prophage (green color region 4), two incomplete prophages (region 2 and region 3 red color), and one questionable prophage (yellow color region 1), as shown in circular map. Figure S7: Multiple-sequence alignment, homology modelling, and prediction of binding pockets of MacB from ECG015 strain. A. Multiple-sequence alignment of *E. coli* MacB and its homologs in bacterial species. The sequences from selected bacteria were aligned using Clustal Omega and visualized in ESPript 3.0. The predicted secondary structure elements for MacB of the ECG015 strain are shown above the alignment. The loop region is indicated in the sequence alignment; arrows represent β-sheets, coils indicate α-helices, “TT” denotes β-turns, and “η” represents 3_10_ helices. Conserved residues are highlighted with a red background, and residues conserved among groups are boxed. The MacB proteins from selected bacteria (with locus ID or accession number in parentheses) are as follows: *E. coli* ECG015 (A8A05_RS17150); *E. coli* (MHO06927.1); *E. coli* (strain K12) (b0879, JW0863); *E. coli* UTI89 (EOV3455025.1; ACONK8_002114); *E. coli* CFT073 (EOC7022007.1); *Acinetobacter baumannii* SDF (ABAYE2984); *A. baumannii* AYE (ABAYE3248); *Klebsiella pneumoniae* NTUH-K2044 (KP1_1880); *K. pneumoniae* (EA160_03430); *Neisseria gonorrhoeae* (Q5MK06); *Pseudomonas aeruginosa* PAO1 (Q9I190); *Burkholderia pseudomallei* MSHR146 (BBN_4023); *Stenotrophomonas maltophilia* (Smal_1297); *Vibrio parahaemolyticus* (WP_025579551.1); and *Aggregatibacter actinomycetemcomitans* (D11S_1304). (ii) Protein sequence alignment of MacB from different *E. coli* strains. MacB sequences from different *E. coli* strains were retrieved, compared with MacB of *E. coli* ECG015 (indicated by a red line open box), aligned using Clustal Omega, and visualized in Jalview (Supplementary Table S4). Amino acid sequences are shown in single-letter code, with position numbers indicated at the top. Sequence similarities and variations are marked with background colors, and highly conserved amino acids are shown with a blue background. The degree of conservation, quality, and consensus of amino acids among all selected *E. coli* strains are displayed in yellow and black. Protein identifiers and sequence lengths are provided in the left panel. B. Homology modelling and superimposed MacB of *E. coli* ECG015. (i) The SWISS-MODEL-predicted MacB monomer model (green) of *E. coli* ECG015 was superimposed with the three-dimensional MacAB-TolC structure (PDB: 5nik.1.J), with the loop region in the predicted ECG015 MacB model indicated by an arrow. (ii) The predicted monomeric MacB model of the ECG015 strain (pink rose) was superimposed with the three-dimensional structure of *A. baumannii* MacB (green, PDB: 5GKO). (iii) The MacB monomer protein model of *E. coli* ECG015 (magenta) was superimposed with the three-dimensional structure of MacB from *Aggregatibacter actinomycetemcomitans* (brown, PDB: 5LJ7). C. Homology model and prediction of binding pockets in MacB protein. (i) A monomeric model of *E. coli* ECG015 MacB was generated using the SWISS-MODEL server with the template structure (PDB: 5nik.1.J). (ii) The generated MacB model was used for binding pocket analysis via the ProteinPlus server (http://proteinsplus.zbh.uni-hamburg.de (accessed on 3 November 2024)). Binding sites were predicted using the DoGSiteScorer tool, and the identified pockets were displayed using a color gradient from high to low druggability scores. The probable druggable binding pocket is highlighted in yellow (P_0), followed by violet (P_1), dark green (P_2), pink (P_3), blue (P_4), light green (P_5), and magenta for pocket P_6. Details of the binding scores are provided in Table 1. Figure S8: Prediction of binding pockets and homology modelling of CpxR–DNA complex. A. Homology Model and Prediction of Binding Pockets in CpxR Protein. A homology model for CpxR from *E. coli* ECG015 was generated using the SWISS-MODEL server with the template P0AE88.1 (CpxR from *E. coli* K12) (i). The generated CpxR model was subsequently used for binding pocket analysis via the Protein Server Plus tool (http://proteinsplus.zbh.uni-hamburg.de (accessed on 3 November 2024)). Binding sites in CpxR were predicted using the DoG Site Scorer, with the identified binding pockets displayed in a color gradient from high to low drug scores. The most probable druggable binding pocket is highlighted in yellow (ii). B. Structure-based virtual screening of compounds against CpxR. The modeled CpxR protein was employed for structure-based virtual drug screening using MTiOpenScreen and AutoDock programs (https://bioserv.rpbs.univ-paris-diderot.fr/services/MTiOpenScreen (accessed on 27 October 2024)). The screening results were analyzed, and the ligand with the highest binding energy was further subjected to AutoDock simulations within the MTiOpenScreen server. The 2D structures of the ligands were docked with the CpxR protein to generate complex structures through the fastDRH web server (http://cadd.zju.edu.cn/fastdrh (accessed on 27 October 2024)). The resulting ligand–protein interaction models are shown for R428 (i) and Proscillaridin (ii) in overlay density maps. C. CpxR and DNA operator complex model. A protein–DNA docking model was generated, with the top-ranked model illustrating the close interaction between CpxR and the promoter region of the tyrosine kinase Wzc (i). In the same model, CpxR is represented in a density map (ii). The RING analysis of the CpxR (green) and Pwzc (brown) complex reveals the interface region, which is highlighted by a yellow box (iii), along with the probable interacting residues involved in the DNA–protein interaction. References [80,99,100,101] are cited in the supplementary materials.
