# Supplementary material for: Genomic and Functional Characterization of Multidrug-Resistant E. coli: Insights into Resistome, Virulome, and Signaling Systems
Source: Antibiotics (Basel). 2025 Jun 30;14(7):667. doi: 10.3390/antibiotics14070667 (PMC12291844; doi:10.3390/antibiotics14070667)
Supplement: Supplementary file 1 [file antibiotics-14-00667-s001.zip › Suppl Figures Caption.pdf]

Figure S1: A. Distribution of subsystems in *E. coli* ECG015 genome. The RAST server was used to predict the subsystems and associated genes in different subsystem categories in ECG015 genome. B. Gene ontology annotation of *E. coli* ECG015 genome. The functional annotations of predicted genes in ECG015 genome were achieved through gene ontology (GO) annotations. The predicted genes were represented in three GO categories, molecular function, cellular component, and biological process. Each category comprises multiple genes encodes for different cellular functions.

Figure S2: Comparative gene cluster alignment and visualization in *E. coli* genomes. Comparison of gene cluster alignments of different *E. coli* genomes along with *E. coli* ECG015 (indicated with black closed circle). Pan-genome explorer (https://panexplorer.southgreen.fr (accessed on 03 November 2024)) based clinker visualization exhibit comparison of gene clusters of *Etk* (CLUSTER0379 tyrosine protein kinase)—SF2A, *Wzc* (CLUSTER6477 tyrosine protein kinase)—SF2B, *srkA* (CLUSTER2224 stress response kinase SrkA)—SF2C and *prkA* (CLUSTER9004 PrkA family serine protein kinase)—SF2D along with its flanking genes in ECG015 and other *E. coli* pathotype genomes. The genome accession numbers are mentioned in Supplementary Table S1.

Figure S3: Multiple-sequence alignment and phylogeny of *E. coli* protein tyrosine kinases Etk and Wzc. A. Multiple-sequence alignment of protein tyrosine kinases from selected *E. coli* pathovars. The homologs of *E. coli* Etk and Wzc from various *E. coli* pathovars, *Shigella dysenteriae*, *Klebsiella pneumoniae*, and *Acinetobacter baumannii* were selected for multiple-sequence alignment using CLUSTAL Omega and formatted using the ESPript server. The *E. coli* Wzc homologs used for alignment include the following strains: ECG015 (A8A05_16435); FMU073332-ETEC (BLJ80_15220); LF82-AIEC (K8B90_14415); FHI_NMBU_03-ExPEC/IPEC (BXO92_00085); E2348/69-EHEC (E2348C_2202); Sakai-EHEC,STEC (BAB36288.2); C237-04-EAST1 (N7L43_09425); MCJCHV-1-NMEC (DPQ29_09290); MS6192-ExPEC (MS6192_02285); 2013C-4225-STEC (C6997_RS23570); 2015C-3107-STEC (C6N21_RS05990); 05-3629-STEC (C6P66_RS20030); 81009-ExPEC (DNNLJILF_02338); KE26-UPEC (O2W55_14155); SA186-UPEC (CHQ92_03580); UCS1-UPEC (LDM94_11940); CLSC36-UPEC (FK541_10155); UTI89-UPEC (MKD43_11185); *Klebsiella pneumoniae* (SSG20669.1); and *Acinetobacter baumannii* (SST05051.1). The *E. coli* Etk homologs used in this study are ECG015 (A8A05_15275); FMU073332-ETEC (WP_000208639.1); FHI_NMBU_03-ExPEC/IPEC (WP_104934141.1); E2348/69-EHEC (WP_000208660.1); Sakai-EHEC,STEC (NP_309164.1); MS6192-ExPEC (WP_000208650.1); 2013C-4225-STEC (WP_000208650.1); 2015C-3107-STEC (WP_000208650.1); 05-3629-STEC (WP_000208650.1); *Shigella dysenteriae* (EFP9419961.1); and *Klebsiella pneumoniae* (CDK77046.1). The accession numbers for each strain are provided in the parentheses. The *E. coli* pathotypes are indicated before each strain name, and the accession numbers are provided in brackets. In the alignment, the highly similar regions are highlighted with a colored background, conserved residues across groups are boxed, and the consensus sequences are displayed below the alignment. B. Phylogenetic relationship of *E. coli* protein tyrosine kinases. A phylogenetic tree was generated using iTOL for the different Etk and Wzc proteins, which were represented in the multiple-sequence alignment. The Etk and Wzc proteins are segregated into two prominent clusters.

Figure S4: Phylogenomic analysis of *E. coli* tyrosine kinases. A. Phylogenetic distribution of Etk and Wzc from *E. coli* strains. The Etk and Wzc protein tyrosine kinases from over 1000 different *E. coli* strains were selected for this study from the Bacterial and Viral Bioinformatics Resource Center (PATRIC) *E. coli* genome datasets. The sequences were aligned using CLUSTAL Omega, and rectangular phylogenetic tree was constructed in iTOL. The Etk and Wzc protein tyrosine kinases were found to be distantly placed and clustered distinctly in the phylogenetic tree. B. Homology models of *E. coli* tyrosine kinase proteins Etk and Wzc. Protein homology models for Etk and Wzc were generated using the respective protein sequences from the *E. coli* ECG015 genome on the SWISS-MODEL server (https://swissmodel.expasy.org (accessed on 27 September 2024, 26 October 2024)). The generated structural models for Etk and Wzc exhibited 99.86% sequence identity with template Q8XC28.1, a tyrosine-protein kinase Etk AlphaFold DB model (ETK_ECO57, *Escherichia coli* O157:H7), and 99.44% identity with template P76387.1, a tyrosine-protein kinase Wzc AlphaFold DB model (WZC_ECOLI, *Escherichia coli* K12), respectively. The modelled proteins, Etk (in blue) and Wzc (in magenta), along with their superimposition, were visualized using PyMOL.

Figure S5: Distribution of adhesins in *E. coli* strains. Heatmap showing the distribution of adhesin genes in ECG015 strain and other *E. coli* pathotypes. The *E. coli* genomes were BLAST searched against reported adhesins and fimbriae systems in adhesiomeR (https://adhesiomer.quadram.ac.uk/app/adhesiomeR (accessed on 13 May 2025)). A heatmap of hierarchically clustered genes with a dendrogram was generated from adhesiomeR data for *E. coli* genomes in Morpheus (https://software.broadinstitute.org/morpheus (accessed on 13 May 2025)). Blue and red color in the heatmap indicates absence and presence of genes.

Figure S6: Prediction of prophage sequences in *E. coli* ECG015 strain. The ECG015 genome was analyzed with PHASTEST for the presence of prophage sequence; analysis resulted in one intact prophage (green color region 4), two incomplete prophages (region 2 and region 3 red color), and one questionable prophage (yellow color region 1), as shown in circular map.

Figure S7: Multiple-sequence alignment, homology modelling, and prediction of binding pockets of MacB from ECG015 strain. A. Multiple-sequence alignment of *E. coli* MacB and its homologs in bacterial species. The sequences from selected bacteria were aligned using Clustal Omega and visualized in ESPript 3.0. The predicted secondary structure elements for MacB of the ECG015 strain are shown above the alignment. The loop region is indicated in the sequence alignment; arrows represent β-sheets, coils indicate α-helices, "TT" denotes β-turns, and "η" represents 3_{10} helices. Conserved residues are highlighted with a red background, and residues conserved among groups are boxed. The MacB proteins from selected bacteria (with locus ID or accession number in parentheses) are as follows: *E. coli* ECG015 (A8A05_RS17150); *E. coli* (MHO06927.1); *E. coli* (strain K12) (b0879, JW0863); *E. coli* UTI89 (EOV3455025.1; ACONK8_002114); *E. coli* CFT073 (EOC7022007.1); *Acinetobacter baumannii* SDF (ABAYE2984); *A. baumannii* AYE (ABAYE3248); *Klebsiella pneumoniae* NTUH-K2044 (KP1_1880); *K. pneumoniae* (EA160_03430); *Neisseria gonorrhoeae* (Q5MK06); *Pseudomonas aeruginosa* PAO1 (Q9I190); *Burkholderia pseudomallei* MSHR146 (BBN_4023); *Stenotrophomonas maltophilia* (Smal_1297); *Vibrio parahaemolyticus* (WP_025579551.1); and *Aggregatibacter actinomycetemcomitans* (D11S_1304). (ii) Protein sequence alignment of MacB from different *E. coli* strains. MacB sequences from different *E. coli* strains were retrieved, compared with MacB of *E. coli* ECG015 (indicated by a red line open box), aligned using Clustal Omega, and visualized in Jalview (Supplementary Table

S4). Amino acid sequences are shown in single-letter code, with position numbers indicated at the top. Sequence similarities and variations are marked with background colors, and highly conserved amino acids are shown with a blue background. The degree of conservation, quality, and consensus of amino acids among all selected *E. coli* strains are displayed in yellow and black. Protein identifiers and sequence lengths are provided in the left panel. B. Homology modelling and superimposed MacB of *E. coli* ECG015. (i) The SWISS-MODEL-predicted MacB monomer model (green) of *E. coli* ECG015 was superimposed with the three-dimensional MacAB-TolC structure (PDB: 5nik.1.J), with the loop region in the predicted ECG015 MacB model indicated by an arrow. (ii) The predicted monomeric MacB model of the ECG015 strain (pink rose) was superimposed with the three-dimensional structure of *A. baumannii* MacB (green, PDB: 5GKO). (iii) The MacB monomer protein model of *E. coli* ECG015 (magenta) was superimposed with the three-dimensional structure of MacB from *Aggregatibacter actinomycetemcomitans* (brown, PDB: 5LJ7). C. Homology model and prediction of binding pockets in MacB protein. (i) A monomeric model of *E. coli* ECG015 MacB was generated using the SWISS-MODEL server with the template structure (PDB: 5nik.1.J). (ii) The generated MacB model was used for binding pocket analysis via the ProteinPlus server (http://proteinsplus.zbh.uni-hamburg.de (accessed on 03 November 2024)). Binding sites were predicted using the DoGSiteScorer tool, and the identified pockets were displayed using a color gradient from high to low druggability scores. The probable druggable binding pocket is highlighted in yellow (P_0), followed by violet (P_1), dark green (P_2), pink (P_3), blue (P_4), light green (P_5), and magenta for pocket P_6. Details of the binding scores are provided in Table 1.

Figure S8: Prediction of binding pockets and homology modelling of CpxR–DNA complex. A. Homology Model and Prediction of Binding Pockets in CpxR Protein. A homology model for CpxR from *E. coli* ECG015 was generated using the SWISS-MODEL server with the template P0AE88.1 (CpxR from *E. coli* K12) (i). The generated CpxR model was subsequently used for binding pocket analysis via the Protein Server Plus tool (http://proteinsplus.zbh.uni-hamburg.de (accessed on 03 November 2024)). Binding sites in CpxR were predicted using the DoG Site Scorer, with the identified binding pockets displayed in a color gradient from high to low drug scores. The most probable druggable binding pocket is highlighted in yellow (ii). B. Structure-based virtual screening of compounds against CpxR. The modeled CpxR protein was employed for structure-based virtual drug screening using MTiOpenScreen and AutoDock programs (https://bioserv.rpbs.univ-paris-diderot.fr/services/MTiOpenScreen (accessed on 27 October 2024)). The screening results were analyzed, and the ligand with the highest binding energy was further subjected to AutoDock simulations within the MTiOpenScreen server. The 2D structures of the ligands were docked with the CpxR protein to generate complex structures through the fastDRH web server (http://cadd.zju.edu.cn/fastdrh (accessed on 27 October 2024)). The resulting ligand–protein interaction models are shown for R428 (i) and Proscillaridin (ii) in overlay density maps. C. CpxR and DNA operator complex model. A protein–DNA docking model was generated, with the top-ranked model illustrating the close interaction between CpxR and the promoter region of the tyrosine kinase Wzc (i). In the same model, CpxR is represented in a density map (ii). The RING analysis of the CpxR (green) and Pwzc (brown) complex reveals the interface region, which is highlighted by a yellow box (iii), along with the probable interacting residues involved in the DNA–protein interaction.
