# Supplementary figures and images for: Genomic and Functional Characterization of Multidrug-Resistant E. coli: Insights into Resistome, Virulome, and Signaling Systems
Source: Antibiotics (Basel). 2025 Jun 30;14(7):667. doi: 10.3390/antibiotics14070667 (PMC12291844; doi:10.3390/antibiotics14070667)

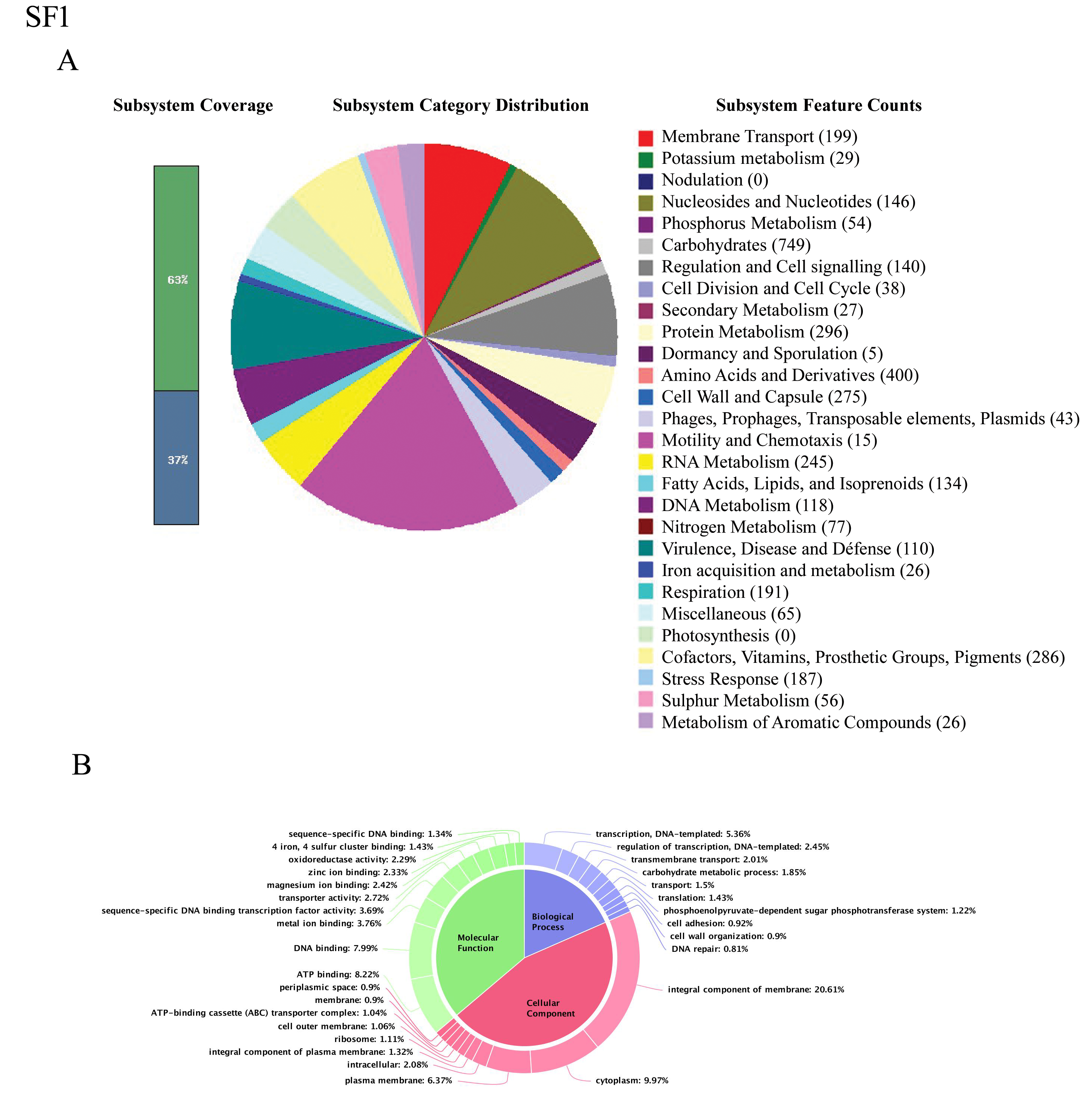

Supplement: Supplementary file 1 [file antibiotics-14-00667-s001.zip › Suppl Figure S1AB.tif]

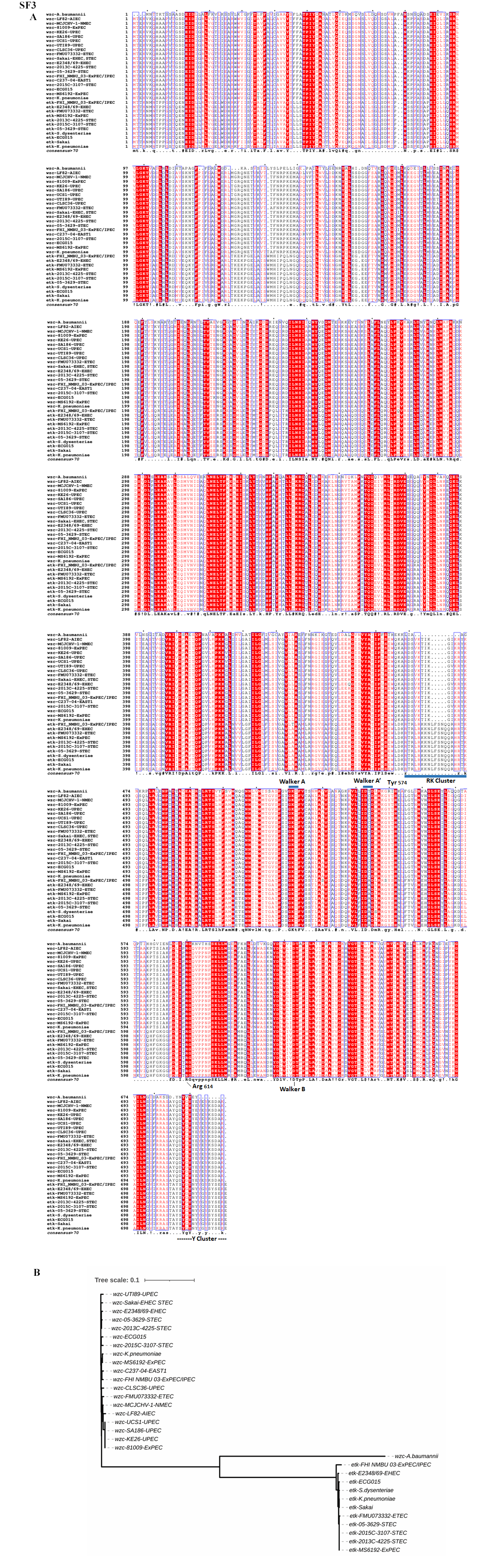

Supplement: Supplementary file 1 [file antibiotics-14-00667-s001.zip › Suppl Figure S3AB.tif]

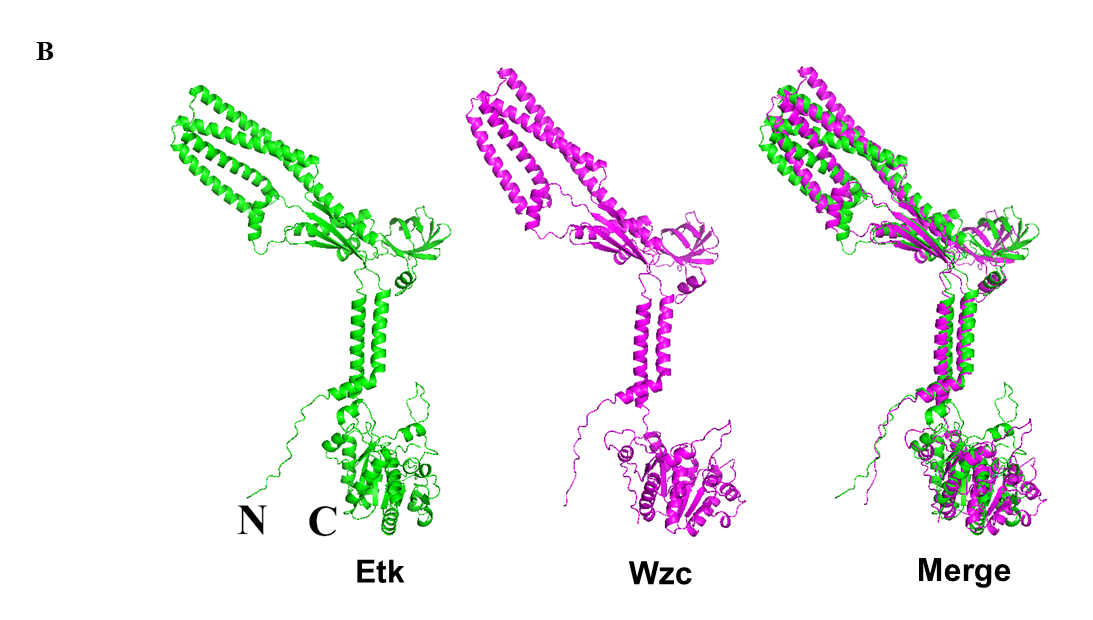

Supplement: Supplementary file 1 [file antibiotics-14-00667-s001.zip › Suppl Figure S4B.TIF]

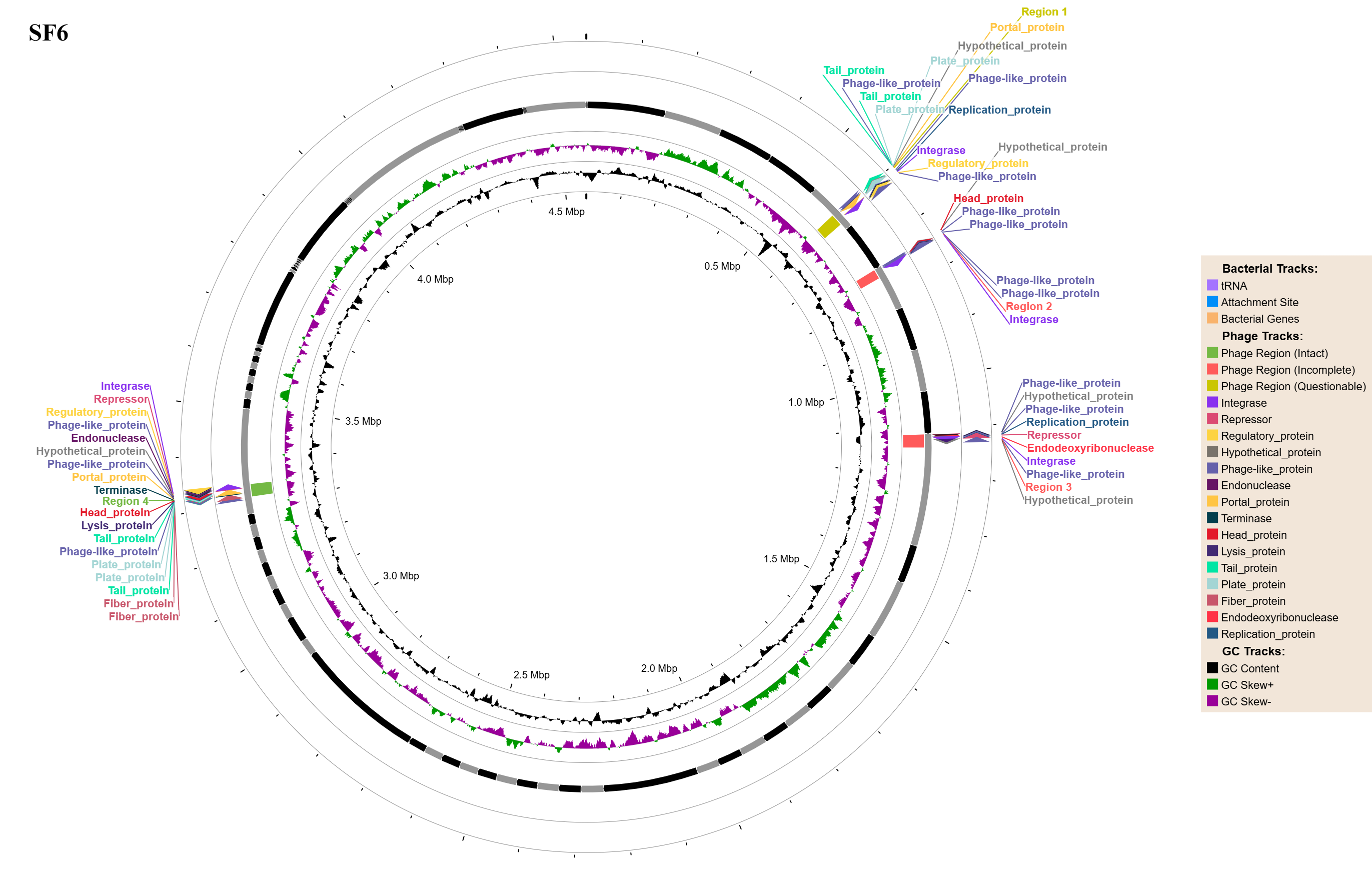

Supplement: Supplementary file 1 [file antibiotics-14-00667-s001.zip › Suppl Figure S6.tif]

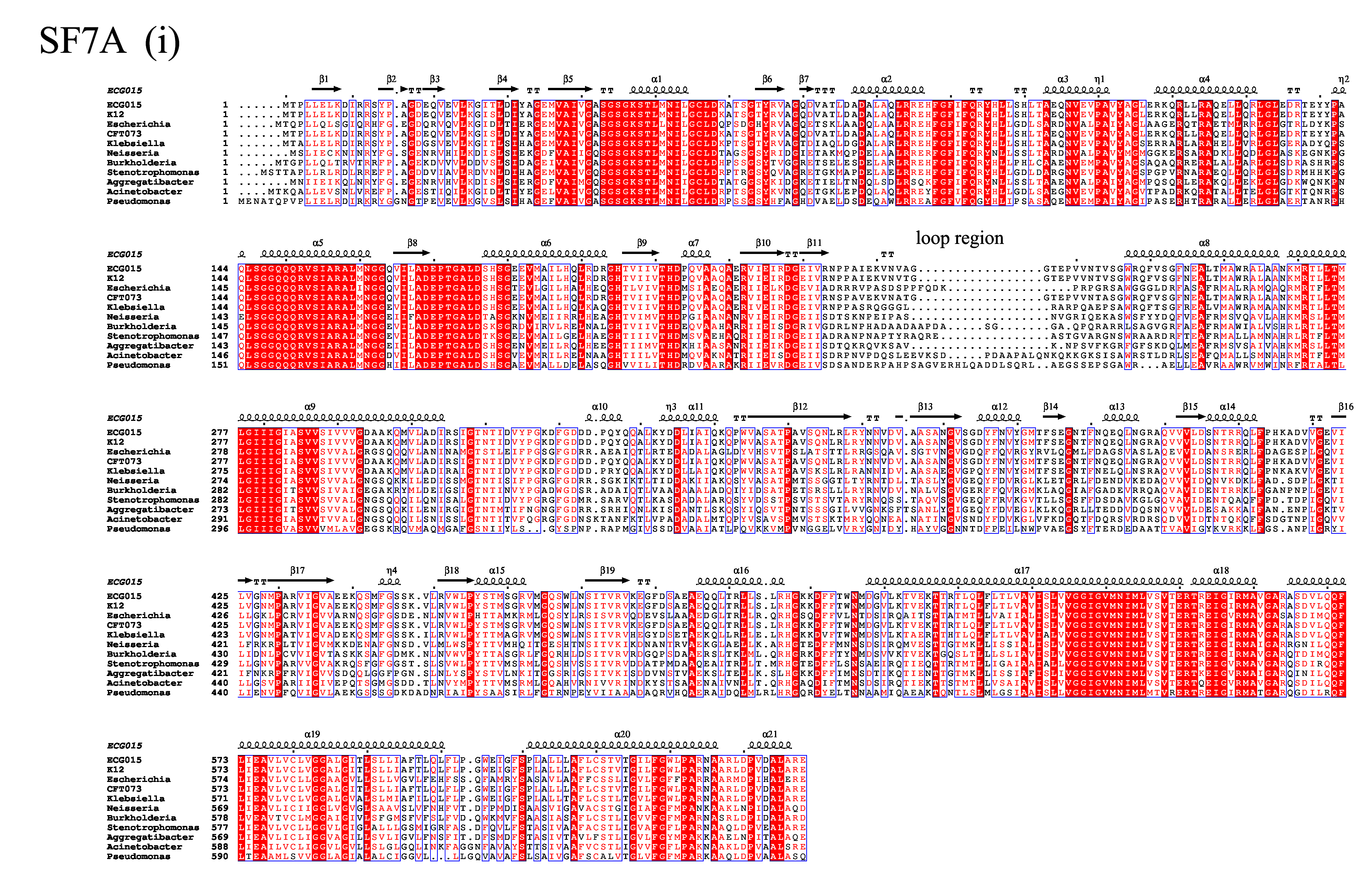

Supplement: Supplementary file 1 [file antibiotics-14-00667-s001.zip › Suppl Figure S7Ai.tif]

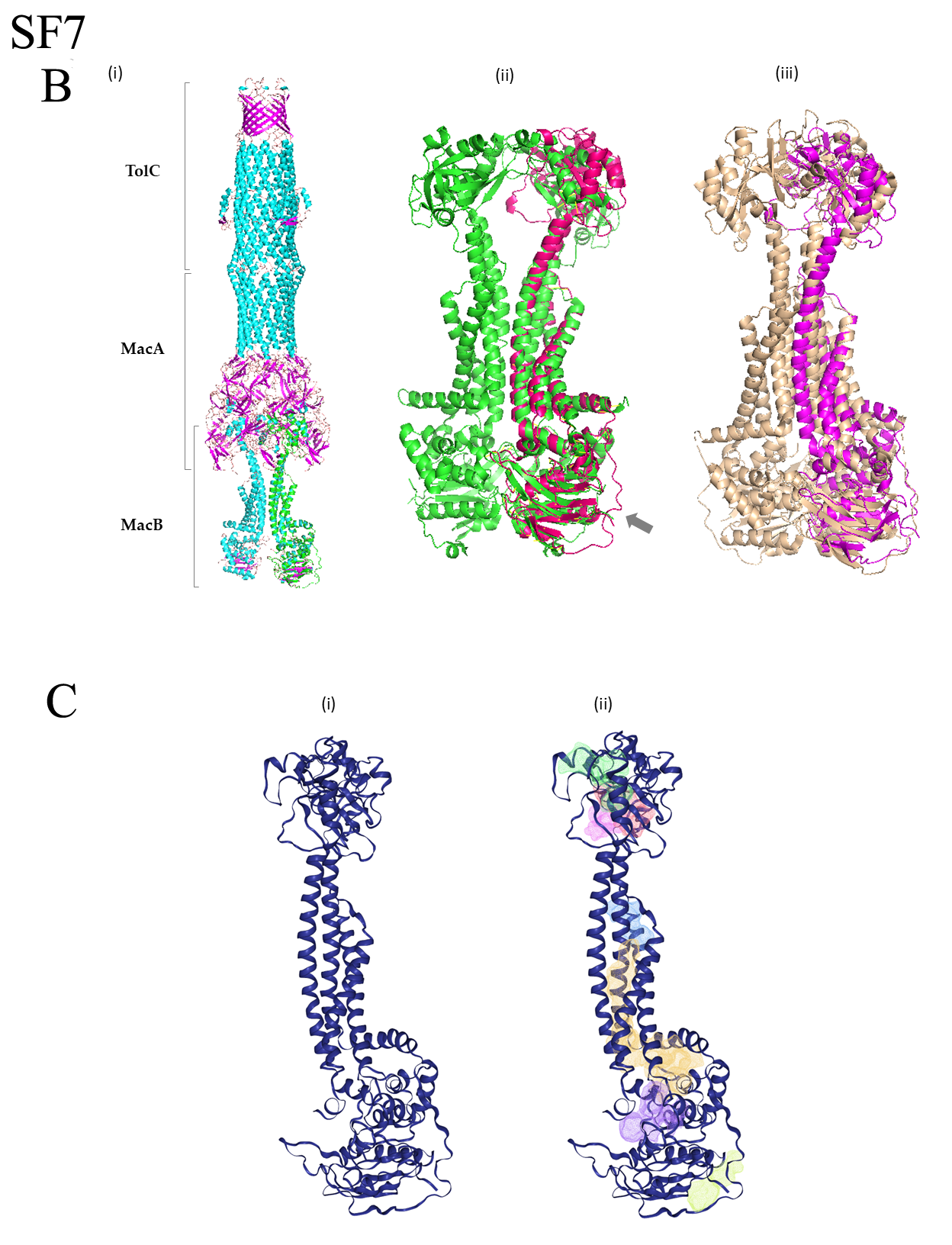

Supplement: Supplementary file 1 [file antibiotics-14-00667-s001.zip › Suppl Figure S7BC.tif]

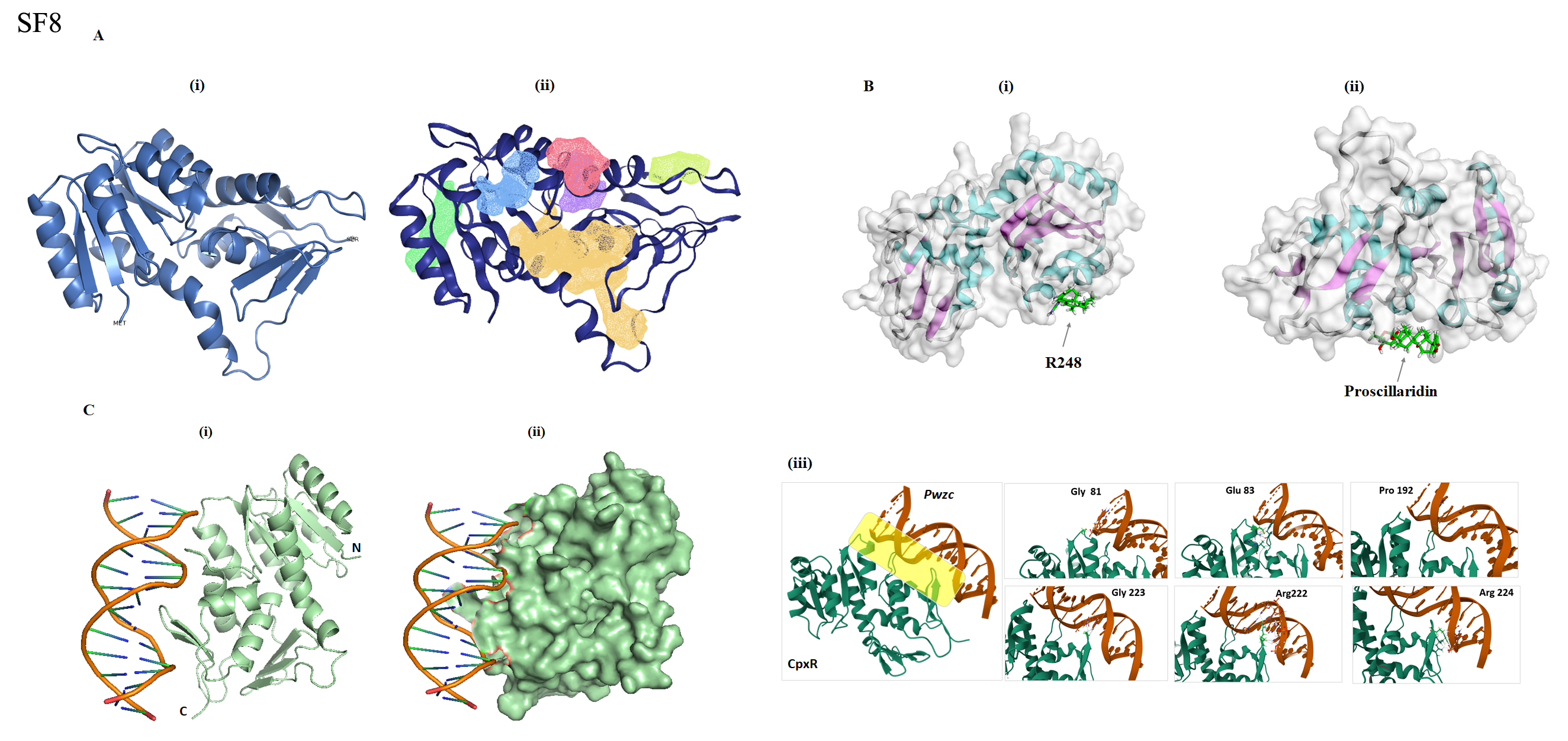

Supplement: Supplementary file 1 [file antibiotics-14-00667-s001.zip › Suppl Figure S8ABC.tif]
