# Supplementary material for: Genomic and Functional Characterization of Multidrug-Resistant E. coli: Insights into Resistome, Virulome, and Signaling Systems
Source: Antibiotics (Basel). 2025 Jun 30;14(7):667. doi: 10.3390/antibiotics14070667 (PMC12291844; doi:10.3390/antibiotics14070667)

SF4A

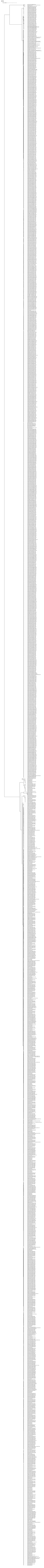

Supplement: Supplementary file 1 [file Suppl Figure S4A.pdf › Suppl Figure S4A.pdf]
